# Anthropogenic interferences lead to gut microbiome dysbiosis in Asian elephants and may alter adaptation processes to surrounding environments

**DOI:** 10.1038/s41598-020-80537-1

**Published:** 2021-01-12

**Authors:** Mohamed Abdallah Mohamed Moustafa, Hla Myet Chel, May June Thu, Saw Bawm, Lat Lat Htun, Mar Mar Win, Zaw Min Oo, Natsuo Ohsawa, Mirkka Lahdenperä, Wessam Mohamed Ahmed Mohamed, Kimihito Ito, Nariaki Nonaka, Ryo Nakao, Ken Katakura

**Affiliations:** 1 Laboratory of Parasitology, Graduate School of Infectious Diseases, Faculty of Veterinary Medicine, Hokkaido University, Kita 18 Nishi 9, Kita-ku, Sapporo, Hokkaido 060-0818 Japan; 2 Department of Animal Medicine, Faculty of Veterinary Medicine, South Valley University, Qena, Egypt; 3 Department of Pharmacology and Parasitology, University of Veterinary Science, Yezin, Nay Pyi Taw 15013 Myanmar; 4 Rector Office, University of Veterinary Science, Yezin, Nay Pyi Taw 15013 Myanmar; 5 Department of Extraction, Myanma Timber Enterprise, Insein, Yangon Myanmar; 6 Sapporo Maruyama Zoo, Sapporo, 064-0959 Japan; 7 Department of Public Health, Turku University Hospital, University of Turku, Turku, Finland; 8 Division of Bioinformatics, Research Center for Zoonosis Control, Hokkaido University, Sapporo, 001-0020 Japan; 9 Present Address: Department of Food and Drug Administration, Ministry of Health and Sports, Zabu Thiri, Nay Pyi Taw 15011 Myanmar

**Keywords:** Conservation biology, Parasitic infection, Microbial communities, Environmental microbiology

## Abstract

Human activities interfere with wild animals and lead to the loss of many animal populations. Therefore, efforts have been made to understand how wildlife can rebound from anthropogenic disturbances. An essential mechanism to adapt to environmental and social changes is the fluctuations in the host gut microbiome. Here we give a comprehensive description of anthropogenically induced microbiome alterations in Asian elephants (n = 30). We detected gut microbial changes due to overseas translocation, captivity and deworming. We found that microbes belonging to *Planococcaceae* had the highest contribution in the microbiome alterations after translocation, while *Clostridiaceae*, *Spirochaetaceae* and Bacteroidia were the most affected after captivity. However, deworming significantly changed the abundance of *Flavobacteriaceae*, *Sphingobacteriaceae*, *Xanthomonadaceae*, *Weeksellaceae* and *Burkholderiaceae*. These findings may provide fundamental ideas to help guide the preservation tactics and probiotic replacement therapies of a dysbiosed gut microbiome in Asian elephants. More generally, these results show the severity of anthropogenic activities at the level of gut microbiome, altering the adaptation processes to new environments and the subsequent capability to maintain normal physiological processes in animals.

## Introduction

Recently, the global loss of biodiversity has negatively affected ecosystems and threatened many wildlife populations^1^. Human activities are considered a leading factor by changing the biological characteristics of ecosystems and causing a loss of organisms, contributing to species extinction^2^. Scientists have tried to understand how animals can recover from these disturbances^3–5^. An essential mechanism to adapt environmental and social changes is the fluctuations in the host gut microbiome, which is important for mammalian host general health including immunity, nutrition and ecological adaptation^6–8^.

The role of gut microbiome can be maintained by several intrinsic factors such as genetic, stress, gender, and age variations. It was demonstrated that several genes are important to maintain microbiome homeostasis in mice such as nitric oxide synthase 2 (NOS2)^9^. In addition, stress-induced gastrointestinal symptoms can occur due to shifts in the gut microbiome in mice^10, 11^. Gender and age have been found to have significant effects on the composition of gut microbiome in several wild mammals such as cheetah^12^ and brown bears^13^. Wildlife-associated microbiota are vulnerable to extrinsic environmental disturbances and the composition and diversity of the gut microbiome in mammals are shaped and maintained by spatial, nutritional, seasonal and social variations^14–17^. Nevertheless, wild animals are continuously captured in huge numbers from their natural habitat for population management, conservation efforts, and zoo exhibition^18^, which can affect their gut microbiome. Previous studies have discussed the effect of captivity on the gut microbiome of wild animals such as Père David's deer^19^ where clear differences in the composition of the gut microbiome were detected between captive and wild individuals. However, little is known about the effect of other anthropogenic interferences on the health of the internal microbiome of wildlife, for example management related activities such as deworming and translocation of animals. In fact, the number of studies that focused on the effects of deworming and translocation on wildlife gut microbiome is very limited due to the restrictive nature of these events. One reason for this limitation is the need to capture and transport of large wildlife for these studies. However, wildlife capturing, and transporting is financially challenging and requiring long time to obtain sufficient sample sizes^20, 21^. In addition, capture and transportation of wildlife will induce stress, compromising the health and welfare of these animals^22^. There is increasing evidence that gut microbiome dysbiosis in humans and animals can lead to inflammatory bowel disease, constipation, colorectal cancer, and malnutrition^23, 24^.

The Asian elephant (*Elephas maximus*) is a notable example of wildlife that are detrimentally affected by anthropogenic activities and is threatened by habitat fragmentation and poaching and is listed as endangered in IUCN Red list since 1986^25^. Previous studies on Asian elephants have indicated differences in the gut microbiota in relation to diet. For example, the reported abundance of Firmicutes and hemicellulose-degrading hydrolases were higher in the gut microbiome from wild Asian elephants than from the captive ones, which suggested that wild elephants are more efficient in digesting lignocellulose^26^. Another study has found that the gut microbial diversity is higher in plant-fed Asian elephants than in breast-fed ones^27^. Although these studies have provided information about the core gut microbiome in this endangered animal, the sample sizes were small (fecal samples from three wild Asian elephants from the Wild Elephant Valley in the Xishuangbanna National Nature Reserve, Yunnan Province, China and two captive Asian elephants from Hagenbecks Tierpark zoo in Hamburg, Germany)^26, 27^. Moreover, these studies did not evaluate the effect of anthropogenic activities on elephant microbiome. In Myanmar, elephants have been used for the logging industries^28^. There are approximately 3,000 Asian elephants owned by the governmental Myanma Timber Enterprise (MTE) and kept under semi-captive conditions allowing elephants to range freely in their natural habitat, with sanctuary and regular veterinary care^29^. To keep their health conditions, the elephants are regularly treated with anthelmintic drugs for deworming since gastrointestinal parasites are common cause of enteritis especially in younger elephants^30, 31^.

Previously, Myanmar has been holding the second largest wild elephant populations after India^32^. However, the numbers have declined to range between 1430 and 2065 due to habitat loss and mining^33^. Although the capture of wild elephants was banned in Myanmar in 1990, illegal capturing has continued until now^18^. In the 1990s, approximately 100 wild elephants were illegally exported from Myanmar every year^34^. In addition, about 21% of all Asian elephants in Europe were obtained from timber camps in Asia and about 60% are captured from the wild^35^. Given such findings and the challenges facing Asian elephant conservation and health, we performed a three-way study to clarify the gut microbiome dysbiosis associated with anthropogenic disturbances, more specifically overseas translocation, captivity and deworming. Translocation is known to be stressful event for the elephants^36^ and captive elephants have lower survival and reproductive rates than elephants in in-situ populations^37^. Previous research on humans^38, 39^, Amur tigers^40^ and equines^41^ have concluded that there were deworming-associated changes in the gut microbiome that could be due to indirect effects of parasite removal or the direct effect of the anthelmintic drug on the microbes. Our study benefits from large sample size and time series sampling in a large, long-lived endangered mammal with its gut microbiome rarely studied so far. A total of 16 semi-captive and 14 captive Asian elephants were investigated to evaluate the effect of three anthropogenic interferences on their gut microbiome. This study will help to provide ideas on how to replace or reestablish a normal microbiome in targeted elephant groups. Obtaining a deeper understanding of gut microbiome alterations in wildlife accompanying anthropogenic activities could be helpful in developing new strategies such as microbiota replacement therapies to undo the expected health problems that are likely to arise^42^. More generally, this study has wide potential to advance conservational and management practices to increase animal welfare not only in wild and captive populations of elephants but also in other animals with similar physiology and diet.

## Results

### Fecal samples collection from Asian elephants exposed to several anthropogenic activities

Ninety-one fecal samples were collected from 30 Asian elephants. A total of 7,328,206 raw paired-end reads were obtained from the Illumina MiSeq sequencer. According to the demultiplexing summary the forward and reverse reads were truncated at 300 and 280 bp, respectively. In addition, a total of 15 and 10 bp were trimmed from the forward and reverse primer regions, respectively. The DADA2 quality control analysis resulted in 3,982,258 high quality paired-end reads and classified into 9385 features. One sample was excluded from the analysis due to quality reasons.

**Figure 1:**
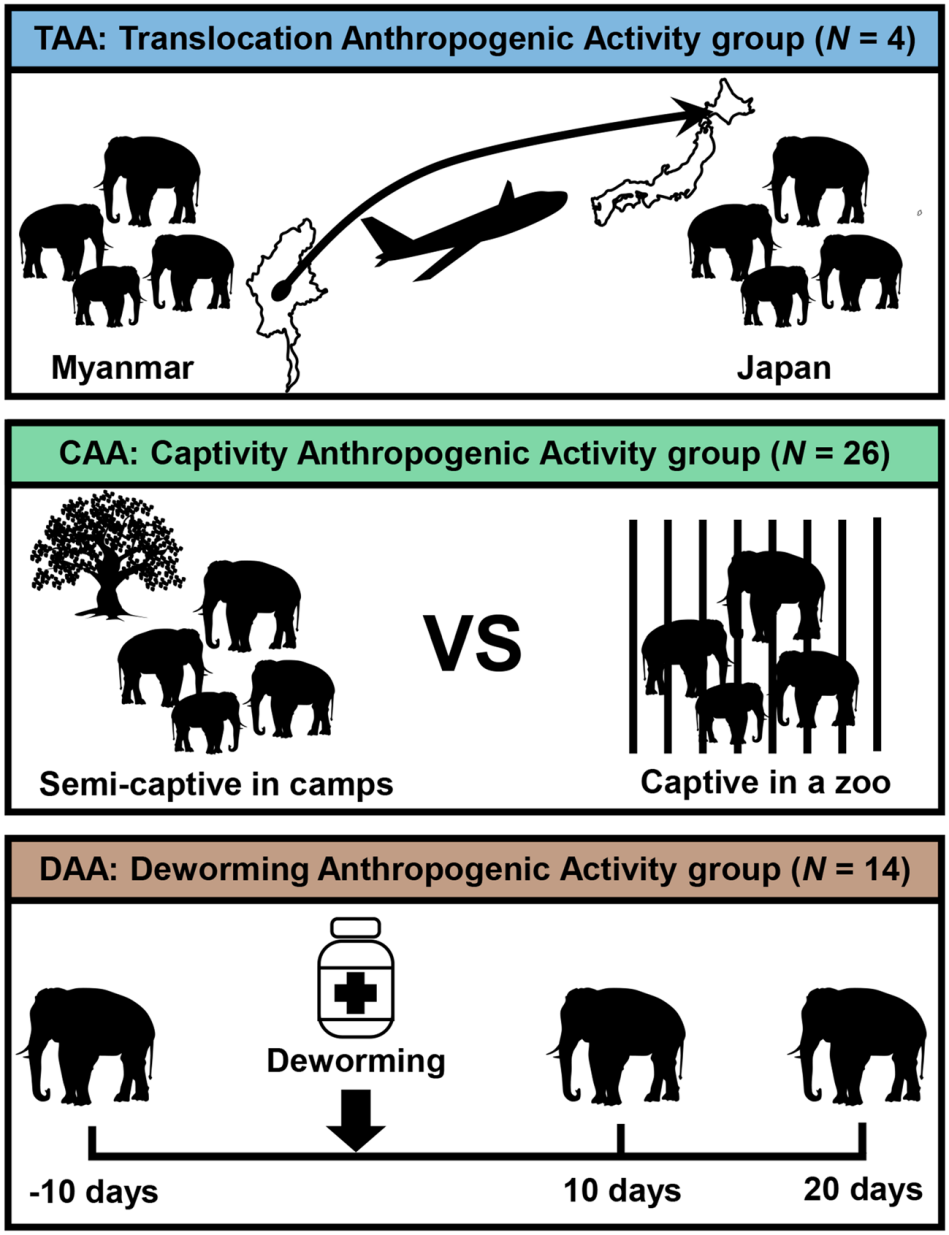
A diagram showing experimental design of each group.

The fecal samples that provided high-quality reads were filtered and divided into three groups. Each group was assigned to study the effect of translocation anthropogenic activity group (TAA), captivity anthropogenic activity group (CAA) or deworming anthropogenic activity group (DAA) on the gut microbiome of Asian elephants (Fig. 1).

### Diversity of gut microbiome from Asian elephants

**Figure 2:**
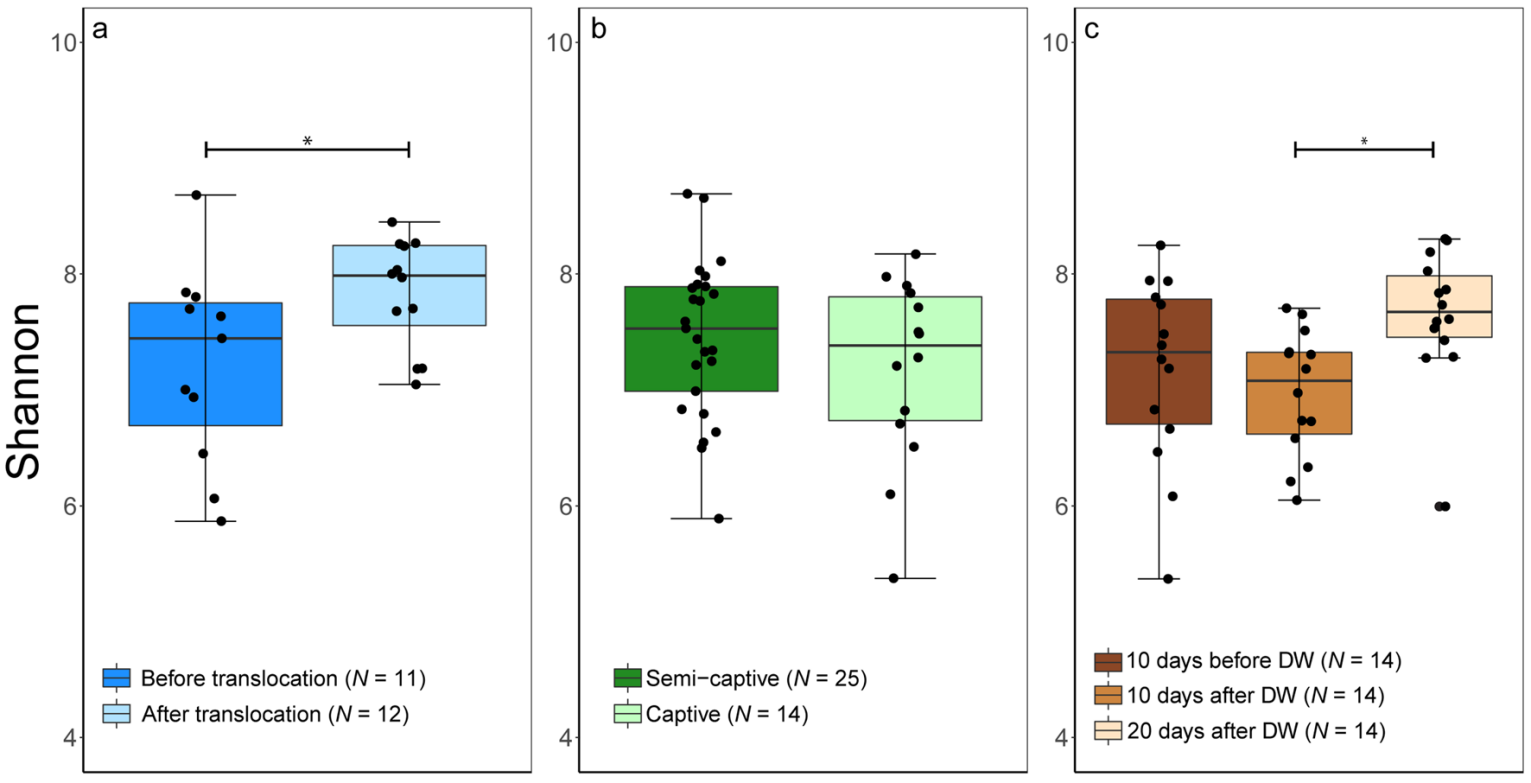
Box and whisker plot describing the alpha diversity comparisons between microbiome communities in Asian elephants. Shannon index was used to quantify the microbiome diversity in elephants before (BT) and after (AT) translocation **(a)**, in captivity and semi-captivity **(b)** and 10 days before, 10 days after and 20 days after deworming **(c)**. *p < 0.05 (Wilcoxon-signed rank test).

**Figure 3:**
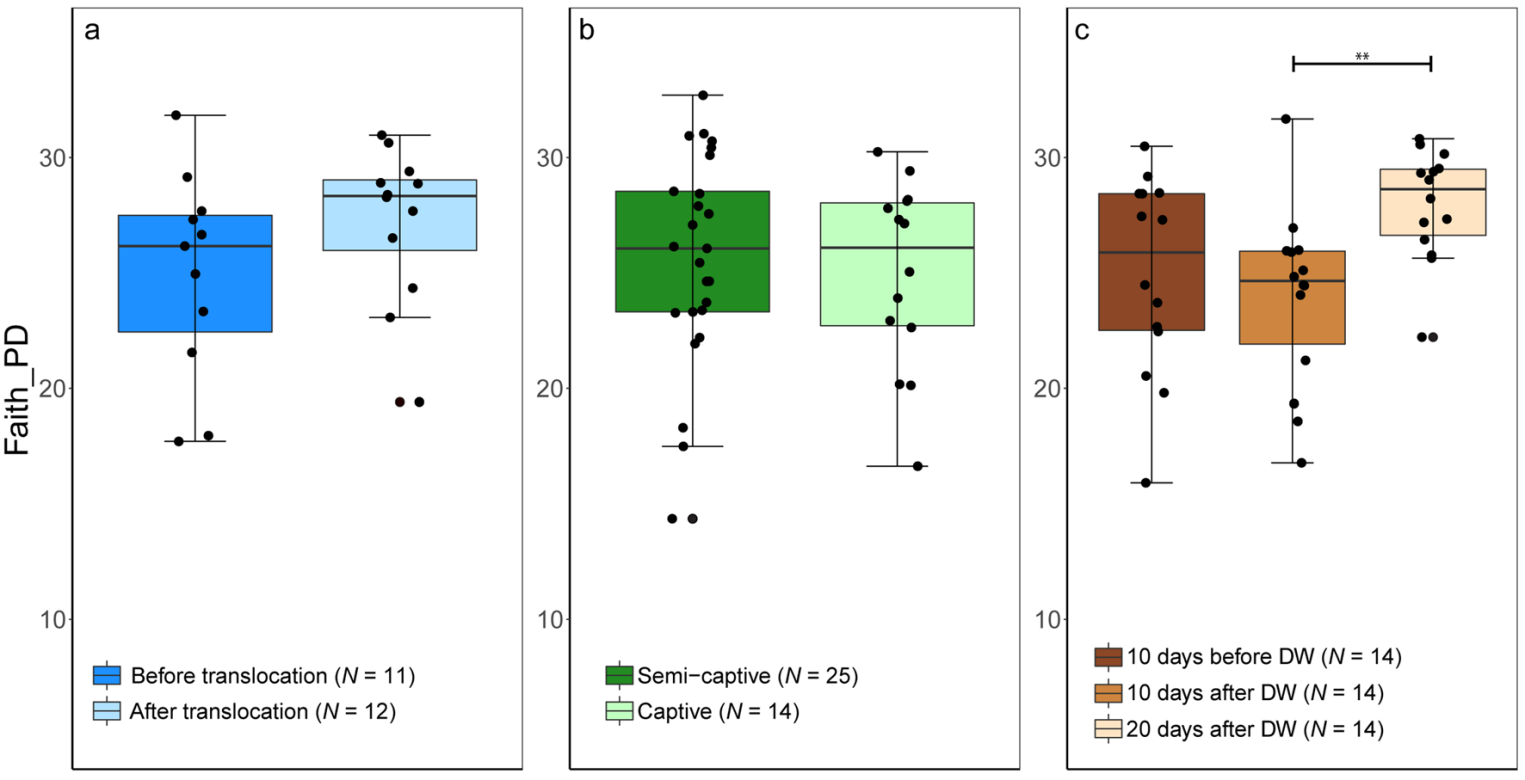
Box and whisker plot describing the alpha diversity comparisons between microbiome communities in Asian elephants. Faith’s Phylogenetic Diversity was used to measure the microbiome diversity and phylogenetic relationships between the features associated with elephants before (BT) and after (AT) translocation **(a)**, in captivity and semi-captivity **(b)** and 10 days before, 10 days after and 20 days after deworming **(c)**. **p < 0.01 (Wilcoxon-signed rank test).

**Figure 4:**
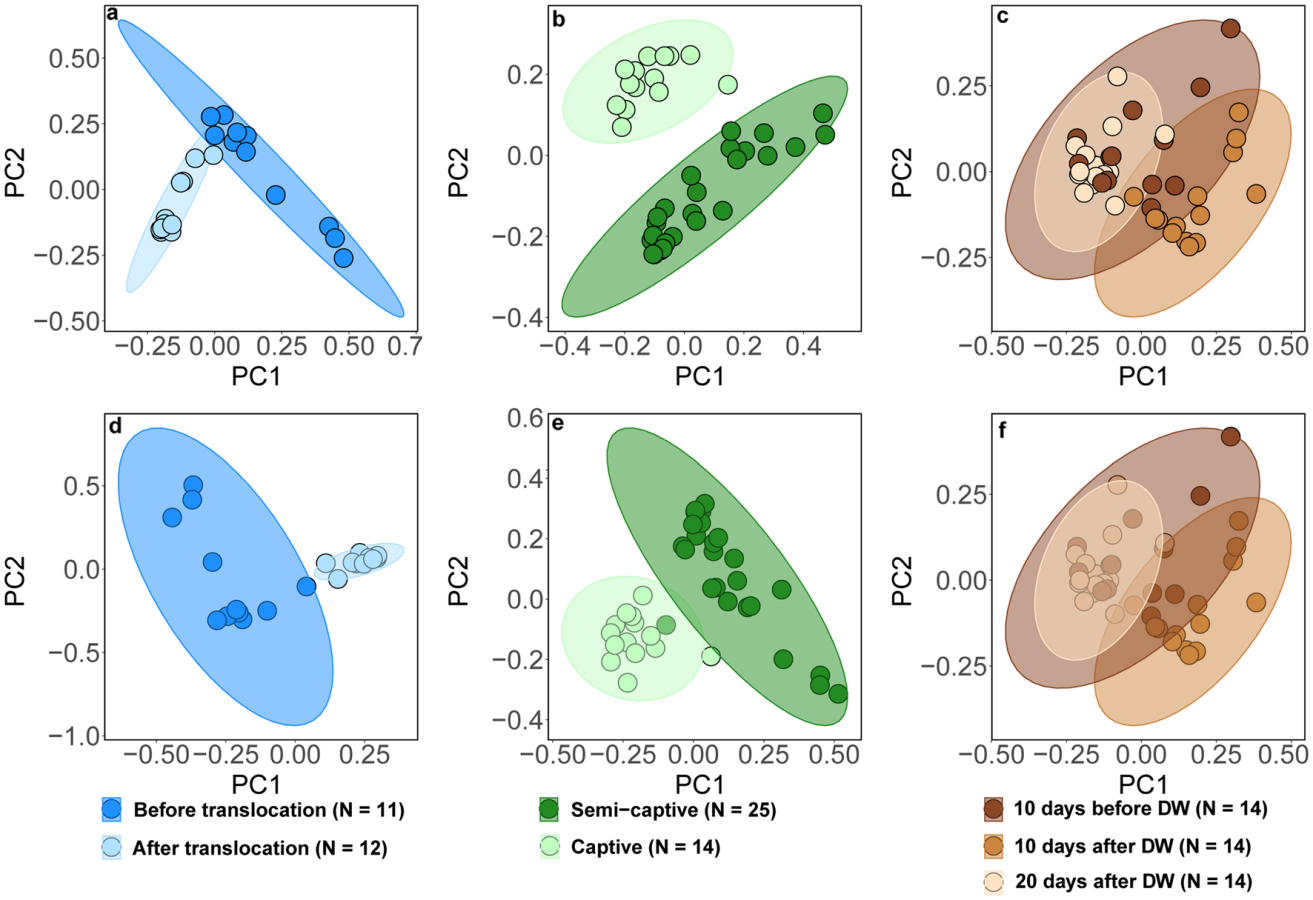
Testing of gut microbiome beta diversity. PCoA plots based on Jaccard (**a–c**) and Bray–Curtis (**d–f**) for samples sequenced using the 16S rRNA gene V3–V4 region. Samples before and after the translocation of elephants are represented in (**a,d)**, samples from individuals living in semi-captivity and in captivity were examined and represented in (**b,e)**, while (**c,f)** show the effect of deworming (DW) on diversity.

The calculated alpha diversity using Shannon and Faith’s Phylogenetic Diversity (Faith’s_PD) analyses showed that Asian elephants are associated with highly diverse gut microbiome where Shannon diversity ranged from 5.4 to 8.7 “mean (SE) = 7.4 (0.1)” and Faith’s_PD ranged from 14.4 to 32.7 “mean (SE) = 25.6 (0.4)” (Figs. 2 and 3). In addition, both beta diversity metrics revealed a distinct clustering of the amplicon sequence variants (ASVs) of the gut microbiome of elephants (clustering coefficient = 0.95) in a response to each anthropogenic activity, translocation (TAA), captivity (CAA) and deworming (DAA) (Fig. 4).

**Figure 5:**
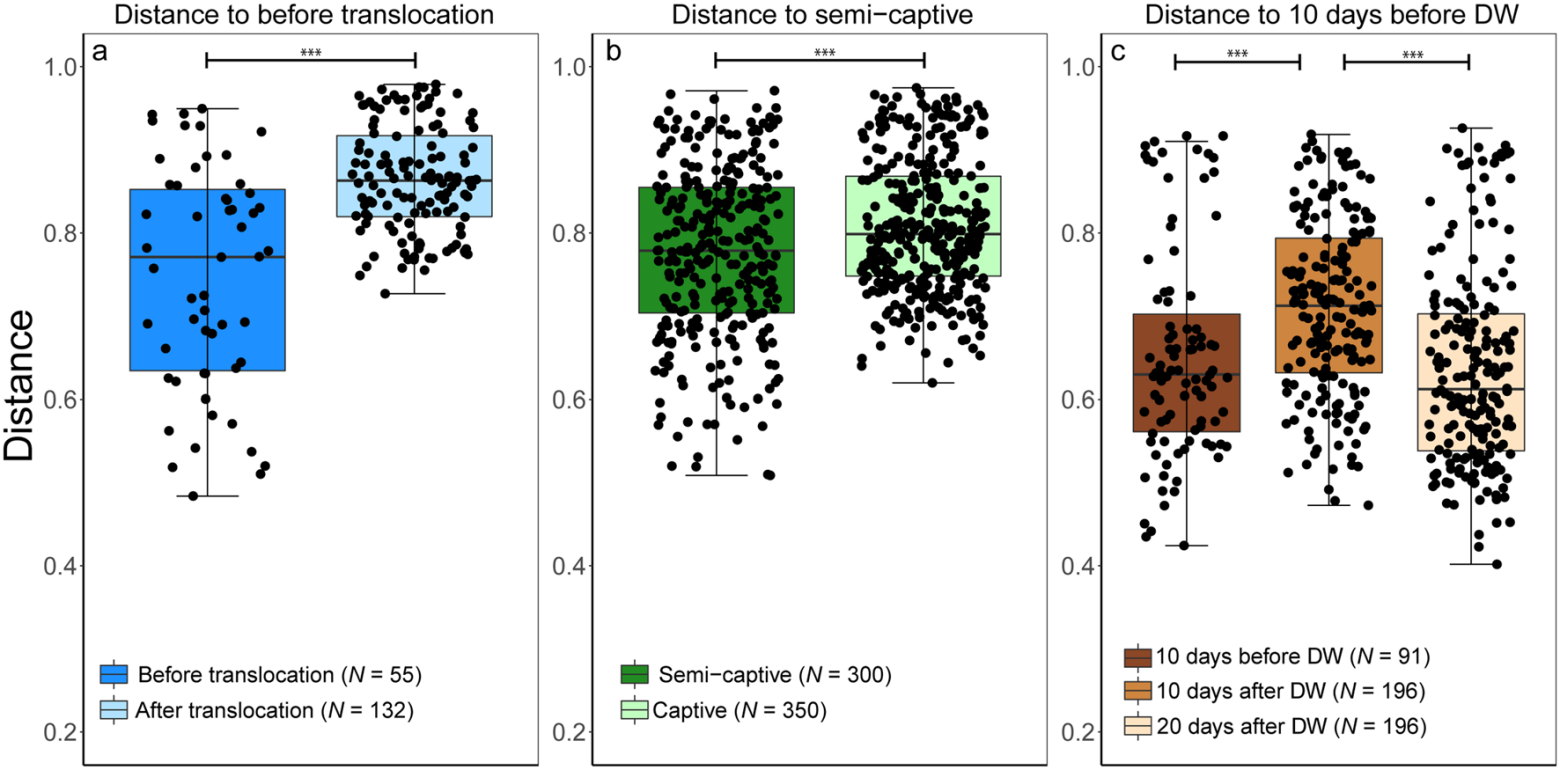
Effect of translocation **(a)**, captivity **(b)** and deworming **(c)** on composition and diversity of the gut microbiome in Asian elephants. Bray–Curtis distances were used to measure the community dissimilarity and analyzed using a pairwise PERMANOVA. *** *p* < 0.001.

Shannon diversity was significantly higher after translocation in TAA group as tested by Wilcoxon-signed rank test (Mean (SE) = 7.5 (0.15), Range = 2.8, and *p* < 0.05) (Fig. 2a). The sequences obtained in 3 successive months from the 4 translocated elephants in Japan clustered together (clustering coefficient = 0.95) and showed lower diversity than before translocation in Myanmar (Fig. 4a,b). The distinct separation of the bacterial communities in Asian elephants associated with translocation was confirmed by a Permutational multivariate analysis of variance (PERMANOVA) that showed significant difference (Permutations = 999, pseudo-F = 5.07, and *p* < 0.001) (Fig. 5a and Supplementary Fig. S1a). However, there were no significant shifts in alpha diversity in CAA group as tested by Wilcoxon-signed rank test for both Shannon diversity (Mean (SE) = 7.4 (0.1), Range = 3.3, and *p* = 0.39) and Faith’s_PD (Mean (SE) = 25.4 (0.7), Range = 18.3, and *p* = 0.59) (Figs. 2 and 3), but the sequences obtained from captive elephants from the zoo in Myanmar clustered together (clustering coefficient = 0.95) and were less dissimilar than those collected from elephants that were living in semi-captivity in the same country (Fig. 4c,d). This was supported by a PERMANOVA test that showed significant difference (Permutations = 999, pseudo-F = 5.23, and *p* < 0.001) in beta diversity of gut microbiome in captive and semi-captive elephants (Fig. 5b and Supplementary Fig. S1b). In addition, the effect of gender and age variations of CAA group on the diversity was not significant as tested by principle coordinates analysis (PCoA) plots based on Jaccard and BC distances (Supplementary Figs. S2 and S3) and Wilcoxon-signed rank test based on Shannon index and Faith’s_PD (Supplementary Fig. S4).

The microbial diversity in DAA group was significantly greater in the samples collected 20 days post deworming as compared to those collected 10 days after, with significance tested by Wilcoxon-signed rank test by both Shannon diversity (Mean (SE) = 7.3 (0.1), Range = 2.3, and *p* < 0.01) and Faith’s_PD (Mean (SE) = 25 (0.7), Range = 14.9, and *p* < 0.01) (Figs. 2c and 3c). In contrast, oral administration of Albendazole 8 mg/kg accounted for changing variations in diversity of gut microbiome across time. PCoA plots based on both Jaccard and BC distances showed a shift of bacterial communities at 10 days after deworming and these communities shifted again to the opposite direction at 20 days after deworming (Fig. 4e,f). The dissimilarity was significant between gut microbiome in elephants before and after deworming (Permutations = 999, pseudo-F = 4.3, and *p* < 0.001, Pairwise PERMANOVA) (Fig. 5c). In addition, a significant difference in beta diversity of gut microbiome was observed in elephants 10 days and 20 days after deworming (Permutations = 999, pseudo-F = 5.9, and *p* < 0.01, Pairwise PERMANOVA) (Supplementary Fig. S1c).

### Gut microbiome structures in Asian elephants

To identify the relationships between each anthropogenic activity and the associated gut microbiomes in Asian elephants, the four most abundant bacterial phyla were compared between groups. The composition of gut microbiome across the examined elephants was dominated by Firmicutes, Bacteroidetes, Proteobacteria and Spirochaetes (Table 1). However, the abundance of these phyla was responded differently to each type of anthropogenic activities as tested by two-sample t-test. For example, the abundance of Proteobacteria (BT: 21.2% and AT: 2.3%) was lower in elephants after translocation in TAA group (t = − 3.9, df = 14.3, *p* < 0.01), while that of Bacteroidetes (BT: 23.1% and AT: 40.3%) (t = 7.3, df = 13.8, *p* < 0.01) and Spirochaetes (BT: 7.3% and AT: 12.1%) (t = 3.4, df = 15.2, *p* < 0.01) increased. In contrast, Firmicutes (10 Days before deworming (10BDW): 43.6%, 10 Days after deworming (10ADW): 30.1% and 20 Days after deworming (20BDW): 46.6%) and Spirochaetes (10BDW: 8.3%, 10ADW: 4.5% and 20BDW: 9.4%) appeared in significantly lower abundance at 10 days post deworming in DAA group than either before deworming (t = − 2.7, df = 24.9, *p* < 0.05 and t = − 2.9, df = 25.2, *p* < 0.01, respectively) or 20 days after (t = − 4.8, df = 22.0, *p* < 0.01 and t = − 4.6, df = 22.0, *p* < 0.01, respectively). The abundance of Proteobacteria at 10 days after deworming was significantly higher (t = 3.7, df = 16.3, *p* < 0.01) than 10 days before deworming (10BDW: 15.9%, 10ADW: 34.1%). While this abundance decreased significantly 20 days after deworming (20BDW: 8.7%) when compared to the abundance of Proteobacteria at 10 days after (t = 6.2, df = 17.9, *p* < 0.01). The observed strong shifts in abundance in TAA and DAA groups in response to translocation and deworming were not present in CAA group in relation to captivity (Table 1).

**Table 1:** The relative abundance of the four most abundant phyla.

Elephant groups	Status	Year	Month	Firmicutes	Bacteroidetes	Proteobacteria	Spirochaetes
TAA	BT	2017	September	40.8%	23.1%^a^	21.2%^b^	7.3%^c^
		2018	January				
		2018	May				
	AT	2018	February	36.8%	40.3%^a^	2.3%^b^	12.1%^c^
		2019	January				
		2019	February				
CAA	Semi-captive	2017	September	42.7%	20.8%	23.3%	7.5%
		2018	January				
		2018	May				
	Captive	2018	February	43.6%	26.0%	15.9%	8.3%
DAA	10 Days before DW	2018	February	43.6%^d^	26.0%	15.9%^e^	8.3%^f^
	10 Days after DW	2018	March	30.1%^d, g^	27.9%	34.1%^e, h^	4.5%^f, i^
	20 Days after DW	2018	March	46.6%^g^	28.7%	8.7%^h^	9.4%^i^

*TAA* translocation anthropogenic activity, *CAA* captivity anthropogenic activity, *DAA* deworming anthropogenic activity, *BT* before translocation, *AT* after translocation, *DW* deworming.

The percentile abundances with the same superscript letter are significantly different (two-Sample t-test, *p* < 0.01).

**Figure 6:**
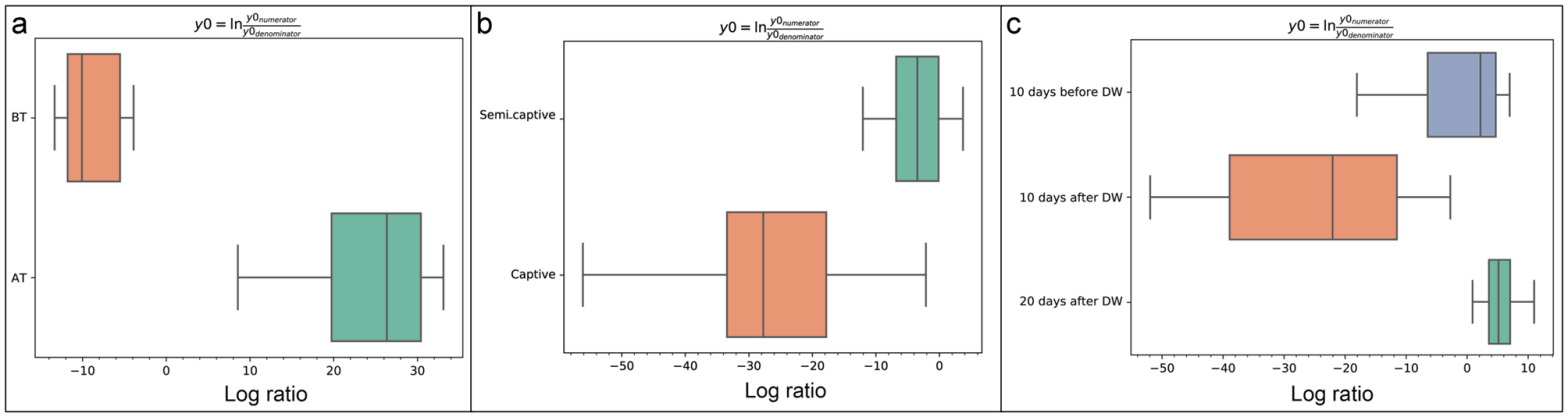
Log ratio of balance of taxa by gneiss analysis of the gut microbiome of Asian elephants.** (a)** Balance of taxa in microbiome before (BT) and after (AT) translocation. (**b)** Balance of taxa in captive against semi captive elephants. (**c)** Balance of taxa in relation to deworming (DW) status. Lower values represent a change in the balance toward denominator taxa, while the higher values represent a change toward numerator taxa.

**Figure 7:**
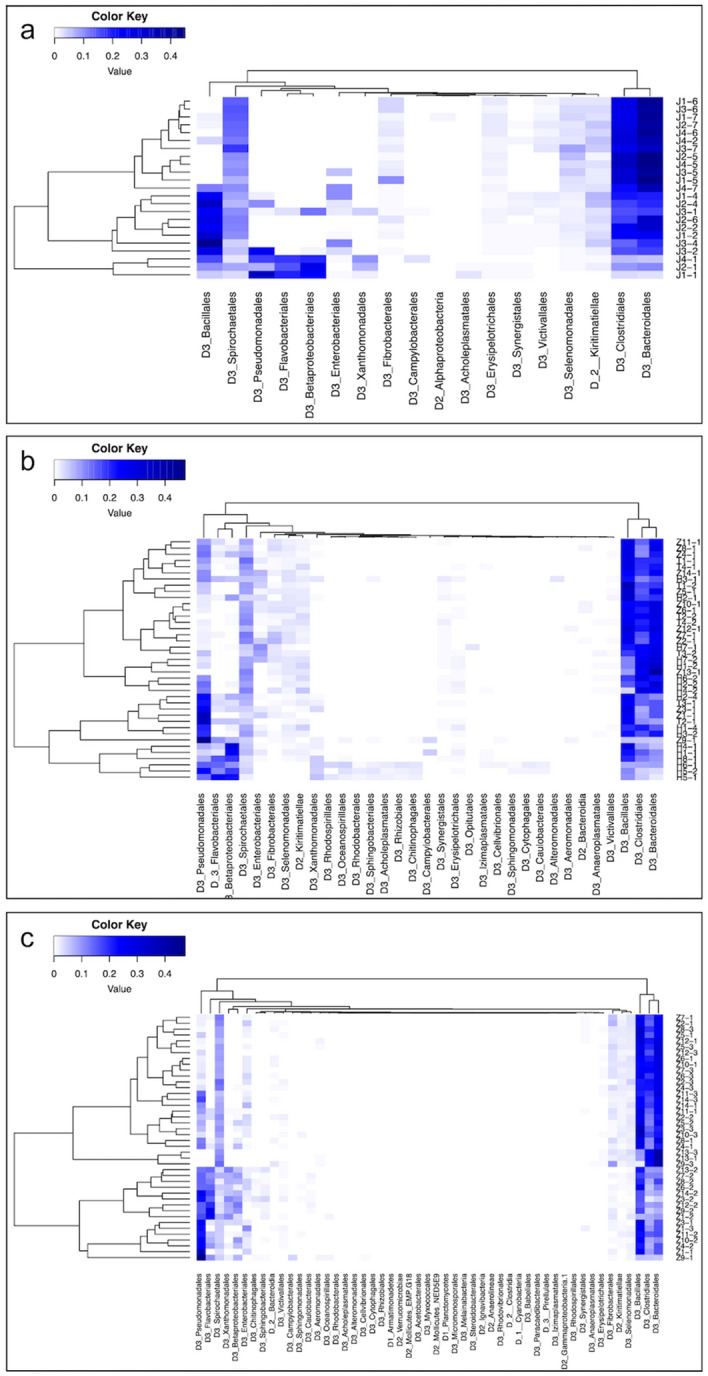
Maximum abundance heatmaps. Alterations in relative abundances of the annotated taxa orders in the gut microbiome of Asian elephants in relation to (**a)** translocation, (**b)** captivity and (**c)** deworming. Elephant IDs and sample numbers are shown on the right side of the heatmaps. Sample IDs are identified by letters that describe the origin of each elephant where (J) represents TAA group from Hmaw Yaw Gyi camp, (H) represents DAA group from Hmaw Yaw Gyi camp, (T) represents DAA group from Taung Kya camp and (Z) represents elephants from Nay Pyi Taw Zoo in Myanmar.

Likewise, the balance of taxa and proportion plots by gneiss analysis revealed that the average log ratios of the detected taxonomic groups increased in TAA group after translocation (y0 _numerator_ taxa = 2403, y0 _denominator_ taxa = 2189), in CAA group in captivity (y0 _numerator_ taxa = 5334, y0 _denominator_ taxa = 866) and in DAA group at 20 days after deworming (y0 _numerator_ taxa = 2032, y0 _denominator_ taxa = 3147) as compared to their respective controls (Fig. 6a–c & Supplementary Figs. S5–S8). The results of analysis of composition of microbiomes (ANCOM) showed that the percentile abundance of *Planococcaceae* (for example: *Lysinibacillus*) was significantly higher before translocation in TAA group (clr = − 6.2, W = 422). In contrast, the abundance of *Clostridiaceae* (for example: *Sarcina;* clr = 3.1, W = 449, and *Clostridium butyricum*; clr = 2.7, W = 408) and Bacteroidia (clr = 3.1, W = 445) was significantly higher in captivity in CAA group. However, abundance of *Spirochaetaceae* (for example: *Treponema* sp. OC1; clr = − 2.9, W = 444) was higher in semi-captivity individuals. Deworming was predominantly associated with significant alterations of the gut microbiome in elephants. The abundance of *Flavobacteriaceae* (for example: *Flavobacterium,* clr = 31.9, W = 513), *Sphingobacteriaceae* (for example: *Sphingobacterium,* clr = 70.3, W = 495), *Xanthomonadaceae* (for example: *Stenotrophomonas,* clr = 28.6, W = 485), *Weeksellaceae* (for example: *Chishuiella,* clr = 34.8, W = 486) and *Burkholderiaceae* (for example: *Comamonas,* clr = 17.2, W = 467) was significantly higher and lower at 10 and 20 days after deworming, respectively, as compared to 10 days before. A maximum abundance among groups analysis, by Vegan and Myseq packages in R software, was executed for each elephant group. The obtained heatmaps supported the results of ANCOM analysis as shown in Fig. 7a–c.

## Discussion

Recently, the study of gut microbiome has emerged as an important tool for monitoring the general health of mammals^43, 44^. Gut microbiome is known to be important to maintain the host health, immunity and behavior^6–8^, but it is largely unknown how human activities impact gut microbiome in mammals in general, and especially in endangered animals needing fast interventions to increase the health, survival and reproductive rates^45, 46^. This study provides insights to help understand how three different types of anthropogenic interventions alter the gut microbiome in Asian elephants. To our knowledge, this is the first investigation to demonstrate the changes in gut microbiome associated with translocation, captivity and deworming in Asian elephants.

The beta diversity and PCoA analysis showed significant differences and distinct separations among the tested elephants induced by translocation, captivity and deworming. This finding suggests that these three anthropogenic activities have a striking effect on the gut microbiome of Asian elephants. In fact, our results showed significant changes in gut microbiome diversity and composition in Asian elephants across time in association with translocation and deworming. Captivity, however, was only associated with a change in community composition (Fig. 5b and Supplementary Fig. S1a), but not alpha diversity. These results suggest that the gut microbial structure within each elephant at Nay Pyi Taw Zoo is distinguishable from that of elephants at Hmaw Yaw Gyi and Taung Kya camps in Myanmar. Our hypothesis is that some gut bacteria in elephants were replaced by those associated with the other animals in the surrounding environment of captivity. It is worth mentioning that this insignificant difference in alpha diversity between gut microbiome in captive and semi-captive elephants was previously observed by other studies on mammals such as mountain goats^47^ and black rhinoceros^48^, which can support our hypothesis.

In contrast, both alpha and beta diversity of the gut bacterial communities were significantly higher in elephants after translocation to Japan, indicating that bacterial diversity in the gut has increased. There are many physiological changes, such as gut microbiome dysbiosis, that can be induced by shipping and transportation of animals as signs of stress, due to the acoustic stress, changing the light cycles and weather stress^49^, but previous studies have indicated that these alterations normalize within 2–3 days after arrival^50, 51^. The dramatic alteration in the gut microbiome of the elephants in our study persisted for three successive months after translocation, which indicates that the translocated elephants were exposed to a variety of bacterial communities in the surrounding environments during and following their trip from Myanmar to Japan. Although, changing habitat from living in semi-captivity in Myanmar to a zoo in Japan was expected to decrease the microbial richness in the gut due to stress^52^. However, our data showed that new microbes were added to the existing community rather than replacing the original gut microbiome.

Parasitic infections are common among Asian elephants^53^, as is the routine deworming in elephant camps in many Asian countries^54^. However, little knowledge is available about the effect of deworming on the gut microbiome of wildlife, especially elephants. Our study is the first to address the effect of using Albendazole as an anthelmintic drug on the gut microbiome of Asian elephants. The significant changes in alpha and beta-diversities of the gut microbiome due to deworming suggest that the gut microbiome of elephants reacted similarly following Albendazole administration over time. These alterations could be due to the direct effect of the drug on the gut microbiome or related to the removal of some parasites from the gut. A strong point of this study is that the elephants were examined twice after treatment with Albendazole. Consequently, we examined both acute and prolonged effects of Albendazole treatment on the gut microbiome. The significant variations in both alpha and beta diversity of the gut microbiome between the two different time points after treatment indicates that Albendazole has both, direct and indirect, effects on the gut microbiome. This finding is supported by the previous studies that deworming can affect the composition and diversity of the gut microbiome in humans^39, 55^ and other animals^40, 41^. In addition, published literature presented that internal parasites are correlated positively with gut microbiome diversity^56, 57^, which can explain the diversity of the gut microbiome of elephants in this study that decreased significantly at 10 days following deworming and then increased at 20 after. Generally, there is a high chance that re-infection with internal parasites can occur shortly after deworming. This means that the gut microbiome is able to readjust to its original state before deworming given enough time, which would explain the high similarity between samples collected 20 days after deworming and 10 days before deworming. In contrast, beta diversity increased and then decreased significantly 10- and 20-days following deworming. These induced alterations could be attributed to several causes; for example, intestinal parasites secret various bacterial inhibitors into the gut environment, that when removed would allow the proliferation of bacterial species that were previously prevented from reproducing^58, 59^. In addition, there could be individual variations between elephants in their response to Albendazole administration due to having different intrinsic factors such as age and gender that can lead to increased beta diversity of gut microbiome 10 days after deworming.

In general, the precise effects of gut microbiome dysbiosis on the health of wild animals are poorly studied^60^. However, these alterations can threaten the nutritional status of these animals through impairing the digestion process and consequently lowering their survival rates^61^. The gastrointestinal dysfunctions were found to be the most obvious manifestation of gut microbiome alterations in dogs^62^. Moreover, studies on the composition and importance of gut microbiome in humans showed that changing the healthy intestinal microbiome can have direct effect on human health by causing inflammations, immune diseases and neurological disturbances^63^. While wild animal species can be affected differently by gut microbiome dysbiosis due to translocation, captivity and deworming, future studies are needed to clarify the impacts of these alterations on the animal conservation^64^.

Our results showed that the gut microbiome of Asian elephants is highly diversified and dominated by Firmicutes, Bacteroidetes, Proteobacteria and Spirochaetes (Supplementary Figs. S9-S11). The previous two studies on the gut microbiome of Asian elephants in Germany and China^26, 27^ showed that Firmicutes and Bacteroidetes are the dominant phyla. However, the abundance and diversity of Proteobacteria and Spirochaetes in our study were different from those reported in either study. This finding suggests a changeable gut microbiome composition and abundance of various taxa that is related more to the environment than elephant physiology. The dominance of Firmicutes and Bacteroidetes in our results was expected as both are the most abundant phyla in most mammals^65, 66^.

We detected an alteration in gut microbiome that was not related to the anthropogenic activities in TAA group. The composition of the gut microbiome from the samples collected in September was different from those in January and May collected from the same individuals before translocation to Japan. Those in September included high abundances of Pseudomonadales, Betaproteobacteriales and Flavobacteriales (Supplementary Fig. S12). We believe that this variation might be due to the change in diet, because the elephants in the camp range freely and feed seasonal plants and that all three groups microflora are abundant in plants^67^. Significant alterations in the gut microbiome due to diet has been shown in many animal species^68^. In addition, the weather in Myanmar is different in September (rainy season) than in January and May (dry season) which may affect the availability of dietary items. This is supported by the strong relationship between diet, seasonal change and gut microbiome that was observed in wild black howler monkeys (*Alouatta pigra*)^69^.

In our study, ANCOM analysis was implemented to identify ASVs with the highest contribution in the alterations detected in each elephant test group. Interestingly, each human activity has affected the gut microbiome differently. The most affected microbiome abundance observed in TAA group was *Planococcaceae*, which was significantly reduced following translocation. The family *Planococcaceae* belongs to the phylum Firmicutes which represents the major group of bacteria in the gastrointestinal tract of mammals^70–72^. Furthermore, the decline in abundance of the genus *Lysinibacillus*, also from the phylum Firmicutes, but not the family *Planococcaceae*, had the highest contribution to the changes in gut microbial diversity associated with translocation. The surrounding environment change of diet and drinking water, and transportation stress could be responsible for the observed changes. Likewise, the rise in class Clostridia and Bacteroidia abundance in elephants living in captivity could be attributed to the change in diet. We speculate that carbohydrates included in the diet fed to elephants in captivity require more cellulolytic bacteria to digest^27, 73^. Furthermore, we detected five different *Treponema* spp. in the gut microbiome of elephants in the present study including *Treponema pectinovorum*, *Treponema saccharophilum* and three uncharacterized *Treponema* spp. The reasoning behind the higher abundance of only one species (*Treponema* sp. OC1) in elephants living in semi-captivity than those in captivity is difficult to discern. Generally, microbes belonging to *Spirochaetaceae* are important for cellulose digestion^74^. In a previous study, the enzyme activity profile was different among treponemes^75^, suggesting that *Treponema* spp. could have different roles in the digestion process in elephants and that *Treponema* sp. OC1 can be responsible for the breakdown of fibers of the plant materials in the semi-captive environment. A similar finding was observed in black rhinos where the proportions of *Treponema* spp. were higher in wild individuals than in captive ones^48^. The effect of using Albendazole as a deworming agent for elephants has affected the gut microbiome in many ways. The abundance of two microbes belonging to Bacteroidetes (*Flavobacterium* sp. and *Sphingobacterium* sp.) and four microbes belonging to Proteobacteria (*Stenotrophomonas* sp., *Chishuiella* sp., *Stenotrophomonas* sp. and *Comamonas* sp.) increased 10 days after deworming, then decreased significantly 20 days post Albendazole administration. This unprecedented effect of Albendazole on the gut microbiome is different than the effect of the other two human activities in this study. These short- and long-term effects of Albendazole treatment on the gut microbiome were reported in humans^38, 39, 76^.

As most studies on endangered wildlife do not focus on anthropogenic-induced alterations of their gut microbiome, this study provides a rare opportunity to understand the effect of three different anthropogenic disturbances on the Asian elephants. Understanding the changes in host associated gut microbiome in relation to overseas translocation, captivity, and deworming regimes can guide future strategies to conserve threatened animal populations through management and treatment. There are promising new ways to manage gut microbiome dysbiosis in animals through transplantation and administration of probiotics ^77^. Fecal microbiome transplants were considered to be a future direction to treat gastrointestinal related disorders such as inflammatory bowel disease and obesity^78^. However, care should be taken during selecting the healthy donors, preserving and delivering the transplant to the target animals to avoid possible negative effects on the recipients^77^. We encourage conservation practitioners and microbiome researchers dealing with gut microbiome dysbiosis in wild animals to collect fecal samples from the healthy adult individuals and preserve it by freezing for the future management and treatment of gastrointestinal dysbiosis in wild animals. Recently, oral delivery of the fecal microbiome transplant has helped to successfully change the altered gut microbiome of koalas to become similar to that of the wild ones^79^. Another way to deliver microbiome transplants to wildlife can be based on coprophagia^80^. In conclusion, differences in both composition and diversity that were detected between elephant groups, as a response to each human interference, require further investigation to interpret this study’s results in relation to stress hormones and the longevity of large mammals.

## Methods

### Study animals and fecal samples

Fresh fecal samples were collected from three different groups of elephants for three independent experiments (Fig. 1). The TAA group included four elephants from Hmaw Yaw Gyi (HYG) camp in Myanmar, where they lived in semi-captive conditions. The age of this group ranged between 4 and 26 years old and consisted of three females and one male (Supplementary Table S1). Time series-sampling was implemented to confirm the observed alterations in gut microbiome were induced by the human activities including translocation, captivity and deworming, and not the other factors such as age, gender or location. Fecal samples were collected from each individual in this group on six separate occasions; three samplings were conducted in Myanmar in September 2017, January 2018 and May 2018 and this was followed by overseas translocation in November 2018 by a charter flight to Sapporo Maruyama Zoo in Hokkaido, Japan, where additional fecal samples were collected in December 2018, January 2019 and February 2019 (Table 1). Each translocated elephant was fed mainly on timothy grass, apple and carrot at Sapporo Maruyama Zoo (Supplementary Table S2). The CAA group included 14 elephants from Nay Pyi Taw Zoo (NZ) in Myanmar where they lived in full captive conditions and fed mainly on tiger grass, mulato grass and banana fruit (Supplementary Table S2); and an additional eight and four elephants as a comparison from HYG and Taung Kya (TK) camps in Myanmar, respectively, where they lived in semi-captive conditions (i.e., control group). The age of NZ elephants ranged between 4 and 26 years old and consisted of 9 females and 5 males, while the age of HYG and TK elephants was ranged between 7 months and 56 years old and consisted of 9 females and 3 males (Supplementary Table S1). Fecal samples were collected once from the captive elephants in February 2018, while two samples were collected from the semi-captive elephants: once in September 2017 and another in January 2018 (Table 1). One additional fecal sample was collected from two semi-captive elephants from HYG in May 2018. The DAA group included the same NZ elephants (n = 14) where they received a deworming dosage of Albendazole (8 mg/kg) in late February 2018 (Fig. 1). Fecal samples were collected at three time points: 10 days before, 10 days after and 20 days after the deworming event (Table 1).

### DNA extraction

The collected fecal samples were kept on ice until DNA extraction. Approximately 2 g of elephant feces were collected as soon as discharged through the rectum in a plastic container (F.T bottle, FEED Corp., Yokohama, Japan) and mixed with 6 mL of saline. Afterwards, the container was intensely shaken, and the resulting fecal mixture was transferred to a 1.5 mL tube. This was followed by centrifugation at 13,000 rpm for 1 min and the supernatant was discarded. This procedure was repeated to obtain 0.3–0.4 g stool pellet, which were used as fecal material for subsequent DNA extraction. Fecal microbial DNA was extracted using PowerFecal DNA Isolation Kit (MO BIO Laboratories, Inc., Carlsbad, CA, USA) according to the manufacturer’s protocol. DNA concentration was measured using a NanoDrop 2000 (ThermoFisher Scientific, MA, USA) and DNA samples were kept at -80 °C until use.

### PCR and Illumina sequencing of the V3–V4 region of the 16S rRNA gene

The extracted DNA was used as a template for PCR amplification of the hypervariable regions V3-V4 of the bacterial 16S rRNA gene using the Illumina barcoded forward primer: 5′-TCGTCGGCAGCGTCAGATGTGTATAAGAGACAGCCTACGGGNGGCWGCAG-3′; and reverse primer: 5′-GTCTCGTGGGCTCGGAGATGTGTATAAGAGACAGGACTA­CHVGGGTATCTAATCC-3′ as recommended by Illumina (San Diego, CA, USA). The library was prepared using the Nextera Index Kit (Illumina) and sequenced with a MiSeq Reagent Kit v3 (600 cycles) on an Illumina MiSeq device according to the manufacturer’s instructions.

### Data processing and analysis

Sequences were demultiplexed by BaseSpace (Illumina) and the obtained forward and reverse sequences were processed using QIIME 2^81^ (version 2019.10.0) and merged together. The ASVs were quality-checked, corrected and filtered, and a feature table was constructed using DADA2 (version: 2019.10.0) pipeline^82^. The feature table was filtered and separated into three tables representing each group of elephants. Alpha diversity was calculated based on Shannon and Faith’s Phylogenetic Diversity analyses. Wilcoxon-signed rank test was used to estimate the statistical differences in alpha diversities between elephant groups. Furthermore, beta diversity was calculated based on Jaccard and Bray–Curtis distance analyses using QIIME 2. PERMANOVA was performed to test for significant differences in beta diversity^83^. The results of both Wilcoxon-signed rank test and PERMANOVA were visualized with ggplot2 package in RStudio. In addition, visualization of clustering of ASVs according to translocation, captivity and deworming, was performed by a PCoA using EMPeror plugin in QIIME 2^84^ according to the Jaccard and Bray–Curtis distance analyses results.

The feature-classifier^85^ in QIIME 2 was used to classify reads and the taxonomy was assigned using silva-132-99-nb classifier. The taxonomic groups (order level) were exported to heatmap_2 function in the heatplus R package (version 2.13.0) to visualize the differential abundance of the taxonomic groups in relation to each variable. Welch’s two-sample t-test was used to estimate the statistical differences in percentile abundances of the four most abundant phyla between elephant groups.

To examine the effect of each human activity on the gut microbiome of Asian elephants we used gneiss analysis^86^ in QIIME 2 through creating a balance of taxa and proportion plots of variants. Finally, ANCOM^87^ was implemented to identify microbes with the highest contribution in the dissimilarity of gut microbiome in elephant groups.

### Ethics

Sample collection from elephants was approved by the University of Veterinary Science, Ministry of Agriculture, Livestock and Irrigation, and Myanma Timber Enterprise, Ministry of Natural Resources and Environmental Conservation, Myanmar. The sampling procedures were performed in accordance with the guidelines established by the Animal Experiment Committee of the Graduate School of Veterinary Medicine, Hokkaido University (Sapporo, Japan). The study is reported in accordance with the ARRIVE (Animals in Research: Reporting In Vivo Experiments) guidelines for reporting experiments involving animals.

## Supplementary Information

Supplementary Information.

## Data Availability

Raw sequence data have been deposited in DDBJ Sequence Read Archive with an accession number of DRA DRA010202.
