# Supplementary material for: Anthropogenic interferences lead to gut microbiome dysbiosis in Asian elephants and may alter adaptation processes to surrounding environments
Source: Sci Rep. 2021 Jan 12;11:741. doi: 10.1038/s41598-020-80537-1 (PMC7803949; doi:10.1038/s41598-020-80537-1)
Supplement: Supplementary file 1 — Supplementary Information. [file 41598_2020_80537_MOESM1_ESM.pdf]

Supplementary Materials

Title: Anthropogenic interferences lead to gut microbiome dysbiosis in Asian elephants and may alter adaptation processes to surrounding environments

Mohamed Abdallah Mohamed Moustafa[1, 2], Hla Myet Chel[1, 3], May June Thu[1, 9], Saw Bawm[3], Lat Lat Htun[3], Mar Mar Win[4], Zaw Min Oo[5], Natsuo Ohsawa[6], Mirkka Lahdenperä[7], Wessam Mohamed Ahmed Mohamed[8], Kimihito Ito[8], Nariaki Nonaka[1], Ryo Nakao[1*], and Ken Katakura[1]

[1] Laboratory of Parasitology, Graduate School of Infectious Diseases, Faculty of Veterinary Medicine, Hokkaido University, Sapporo 060-0818, Japan

[2] Department of Animal Medicine, Faculty of Veterinary Medicine, South Valley University, Egypt

[3] Department of Pharmacology and Parasitology, University of Veterinary Science, Yezin, Nay Pyi Taw 15013, Myanmar

[4] Rector office, University of Veterinary Science, Yezin, Nay Pyi Taw 15013, Myanmar

[5] Department of Extraction, Myanma Timber Enterprise, Insein, Yangon, Myanmar

[6] Sapporo Maruyama Zoo, Sapporo 064-0959, Japan

[7] Department of Public Health, University of Turku and Turku University Hospital, Turku, Finland

[8] Division of Bioinformatics, Research Center for Zoonosis Control, Hokkaido University, Sapporo 001-0020, Japan

[9] Present Address: Department of Food and Drug Administration, Ministry of Health and Sports, Zabu Thiri, Nay Pyi Taw 15011, Myanmar

*Corresponding author: Ryo Nakao

Dr. Ryo Nakao

Associate Professor

Laboratory of Parasitology, Faculty of Veterinary Medicine, Hokkaido University, Kita 18 Nishi 9, Kita-ku Sapporo, Hokkaido 060-0818, Japan

E-mail; ryo.nakao@vetmed.hokudai.ac.jp

Supplementary Table S1 Description of the Asian elephants that were included in this study. The elephants were divided into translocation anthropogenic activity (TAA), captivity anthropogenic activity (CAA) and deworming anthropogenic activity (DAA) groups. Samples, that were collected before deworming from elephants in DAA, were also used as samples from captive elephants in CAA.

Elephant group	ID	Status	Age (months)	Gender
TAA	J1	Semi captive	312	Female
	J2		48	Female
	J3		168	Female
	J4		108	Male
CAA	H1	Semi captive	72	Female
	H2		96	Male
	H3		144	Female
	H4		132	Male
	H5		660	Female
	H6		180	Female
	H7		7	Female
	H8		156	Female
	T1		432	Female
	T2		348	Female
	T3		36	Male
	T4		60	Female
DAA*	Z1	Captive	288	Male
	Z2		300	Male
	Z3		252	Male
	Z4		312	Female
	Z5		156	Female
	Z6		204	Female
	Z7		228	Female
	Z8		228	Female
	Z9		156	Female
	Z10		96	Female
	Z11		72	Female
	Z12		60	Male
	Z13		48	Female
	Z14		60	Male

* Samples, that were collected from this group of elephants before deworming, were also used to represent elephants in captivity in CAA group.

Supplementary Table S2 Feed items of Asian elephants in captivity

**Nay Pyi Taw Zoo, Myanmar**	**Sapporo Maruyama Zoo, Japan**
Tiger grass	Dried timothy
Mulato grass	Apple
Banana	Carrot
Tamarind	Banana
Salt	Orange and Pineapple
Ground nut cake	Hay, including alfalfa
Wheat bran	Cabbage
Rice	Sweet potato
Chickpea	Banana plant
Corn bran	Sycamore
Banana plant and watermelon (hot season only)	Pellet (including corn, alfalfa, sugar beet, flower, honey)

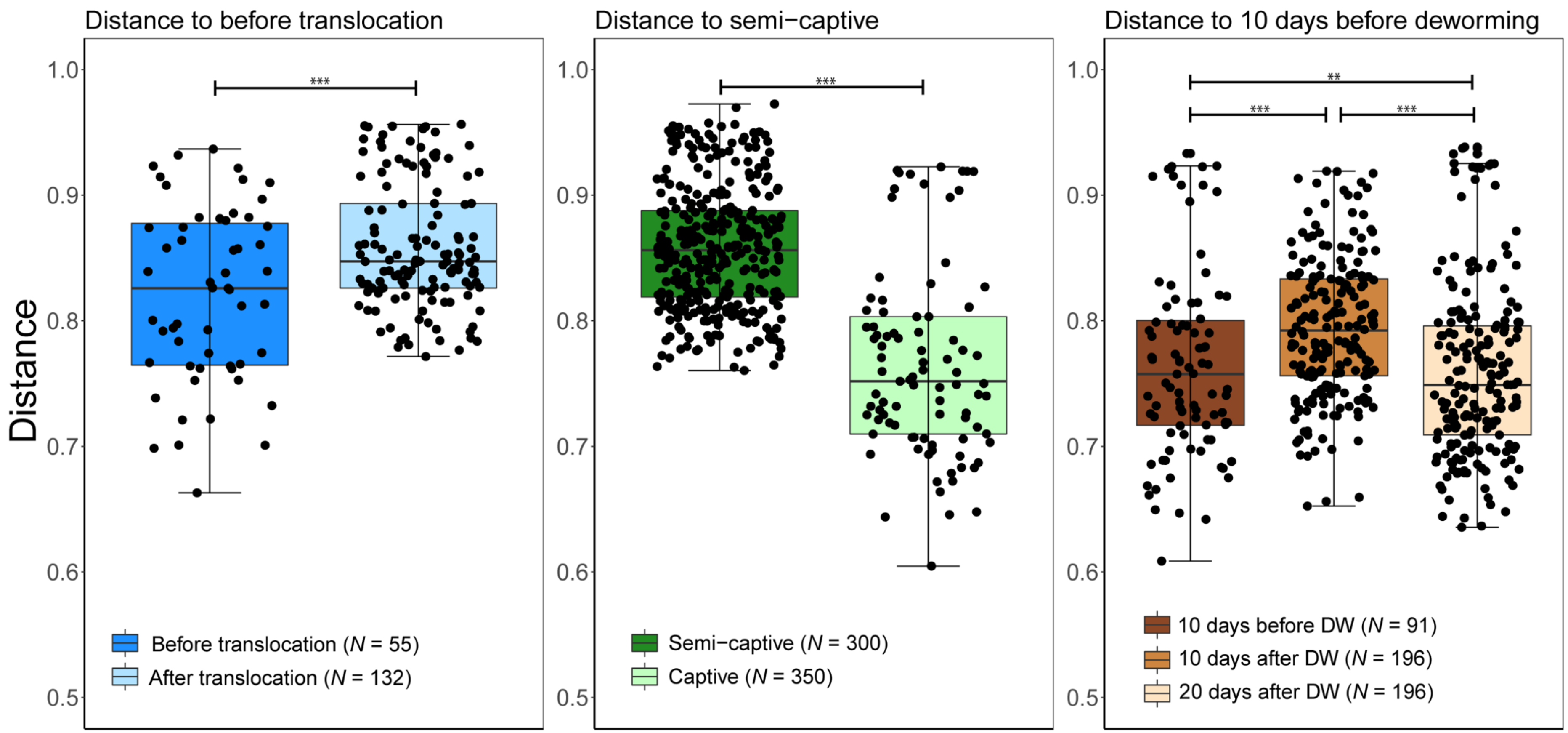

Supplementary Fig. S1 Effect of translocation (a), captivity (b) and deworming (c) on composition and diversity of the gut microbiome in Asian elephants. Jaccard distances were used to measure the community dissimilarity and analyzed using a pairwise PERMANOVA. ** *p-value* < 0.01 and *** *p-value* < 0.001.

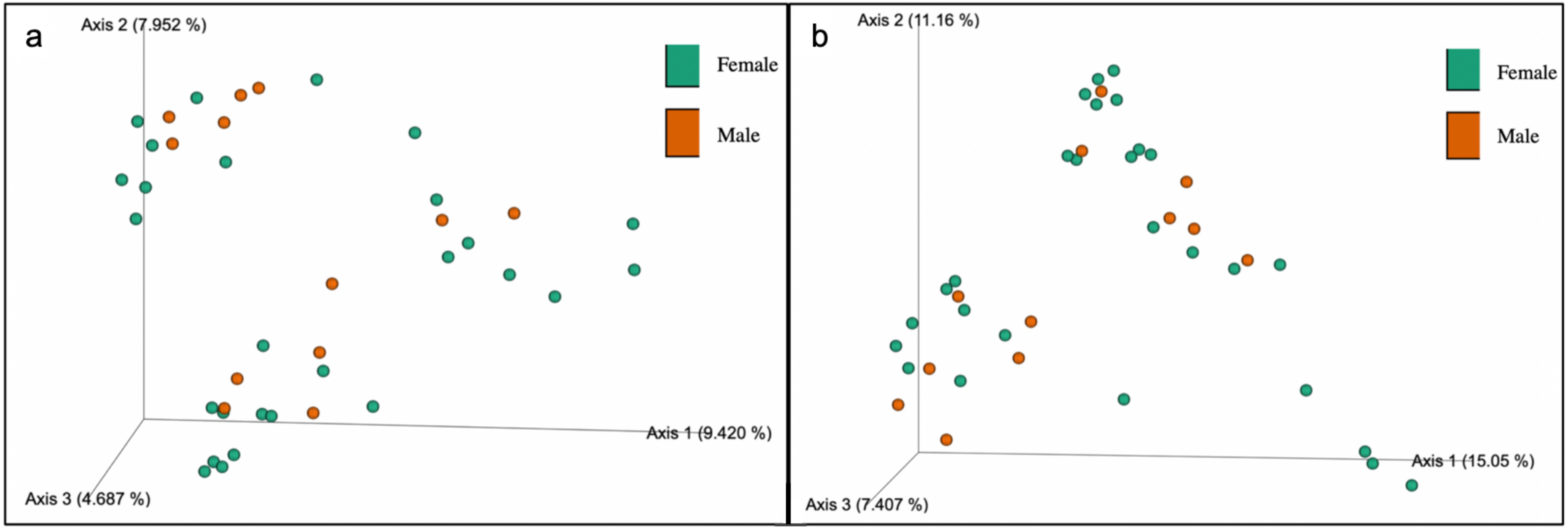

Supplementary Fig. S2 PCoA plots based on Jaccard (**a**) and Bray-Curtis (**b**) for samples sequenced using the 16S rRNA gene V3–V4 region. Samples from CAA group were examined to show the effect of gender variation on diversity.

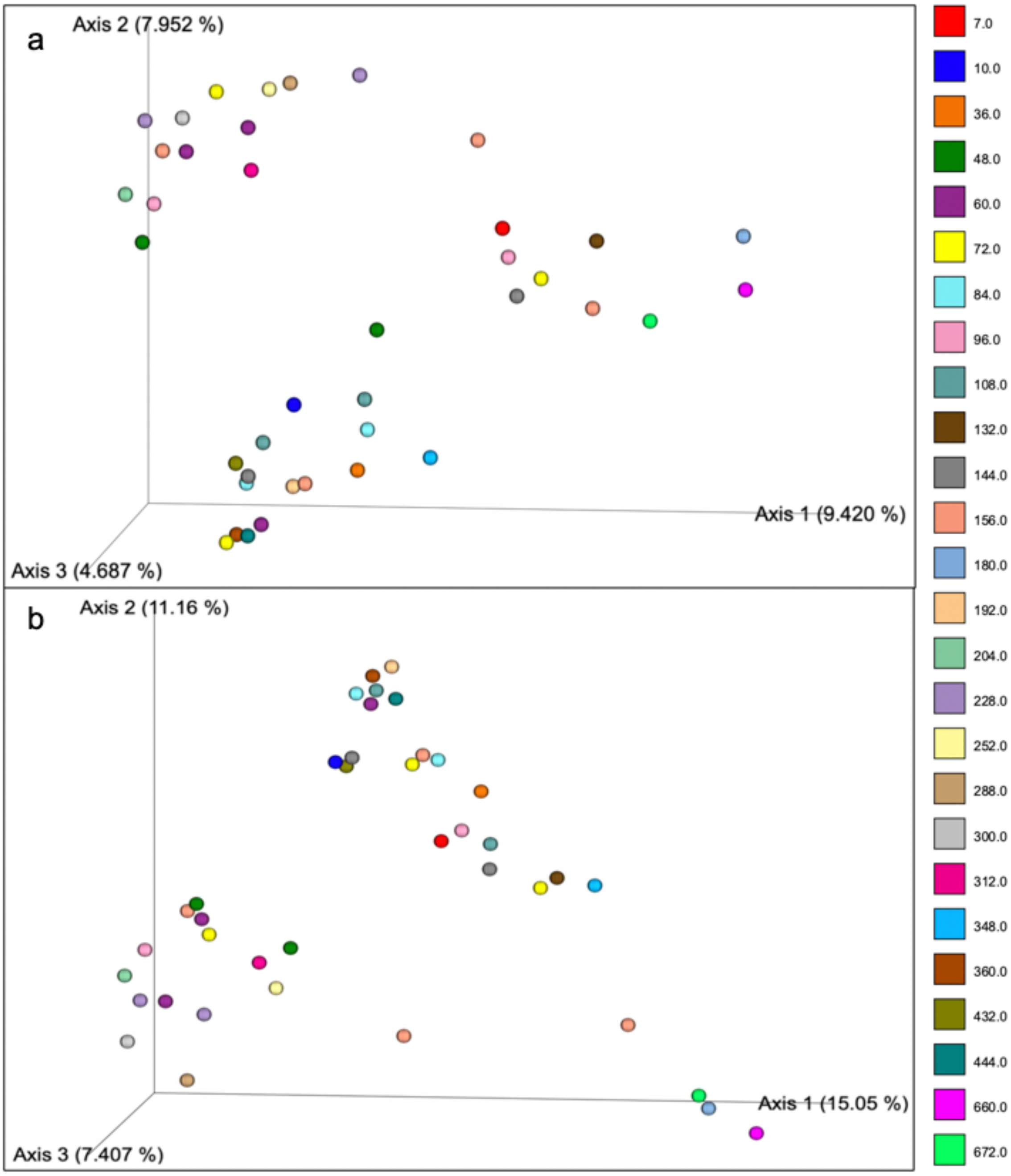

Supplementary Fig. S3 PCoA plots based on Jaccard (**a**) and Bray-Curtis (**b**) for samples sequenced using the 16S rRNA gene V3–V4 region. Samples from CAA group were examined to show the effect of Age (months) variation on diversity.

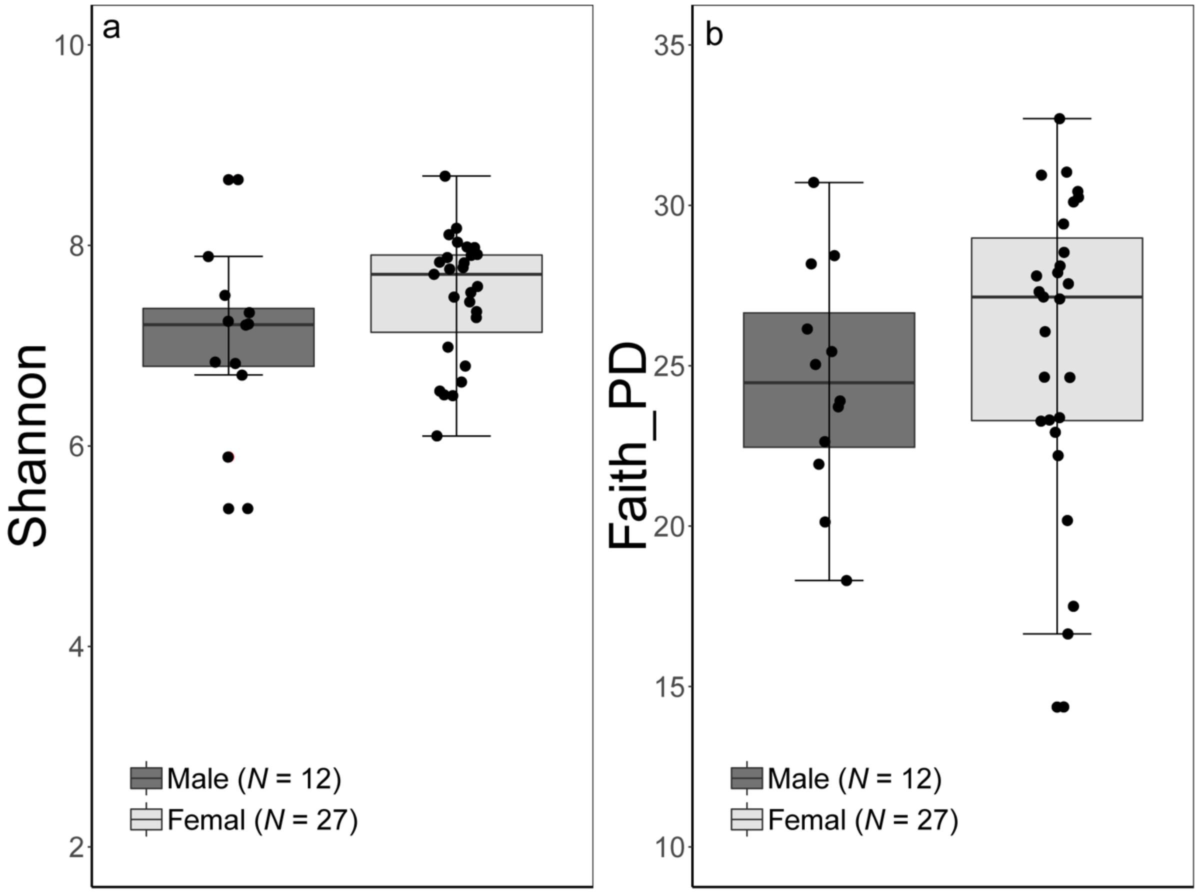

Supplementary Fig. S4 Box and whisker plot describing the alpha diversity comparisons between microbiome communities in Asian elephants. Shannon index (**a**) and Faith's Phylogenetic Diversity (**b**) were used to show the effect of Gender on the gut microbiome diversity in the CAA group. The effect of gender was insignificant by Shannon index (*p-value* > 0.09) and Faith's Phylogenetic Diversity (*p-value* > 0.5) (Wilcoxon-signed rank test).

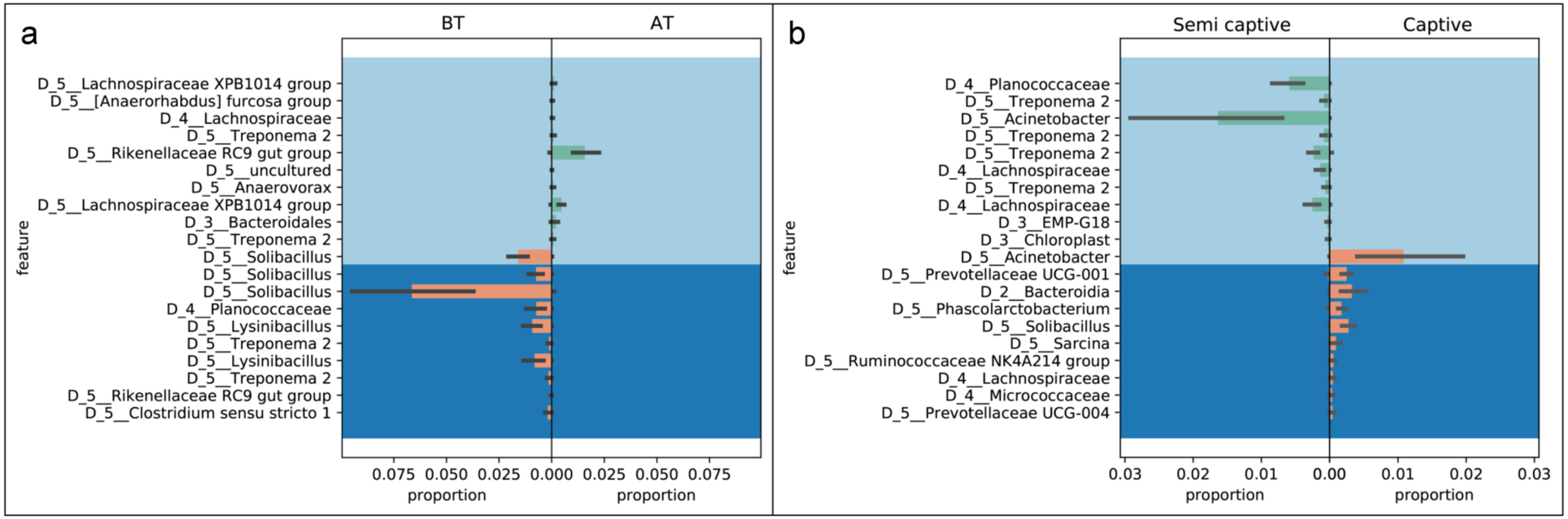

Supplementary Fig. S5 Proportion plots by gneiss representing the differential abundance analysis of balances of taxa in the gut microbiome of Asian elephants **a**: before (BT) and after (AT) translocation and **b**: captive and semi-captive elephants

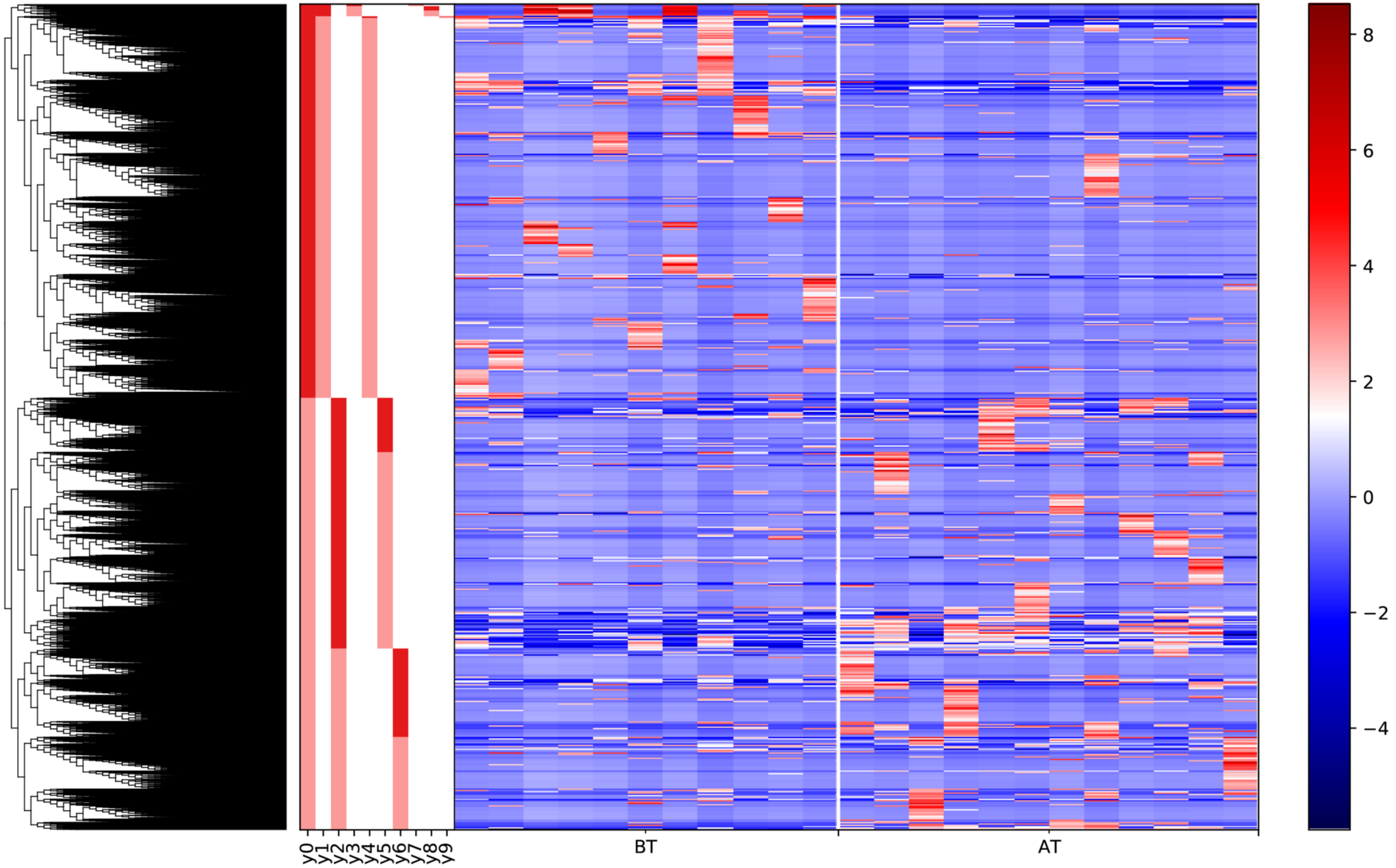

Supplementary Fig. S6 A dendrogram heatmap showing the effect of **translocation** on log abundance of ASVs in the gut microbiota of Asian elephants. Changes in the relative abundance in elephants before (**BT**) and after (**AT**) translocation can be seen in the balance y0, y2 and y6 (gneiss analysis).

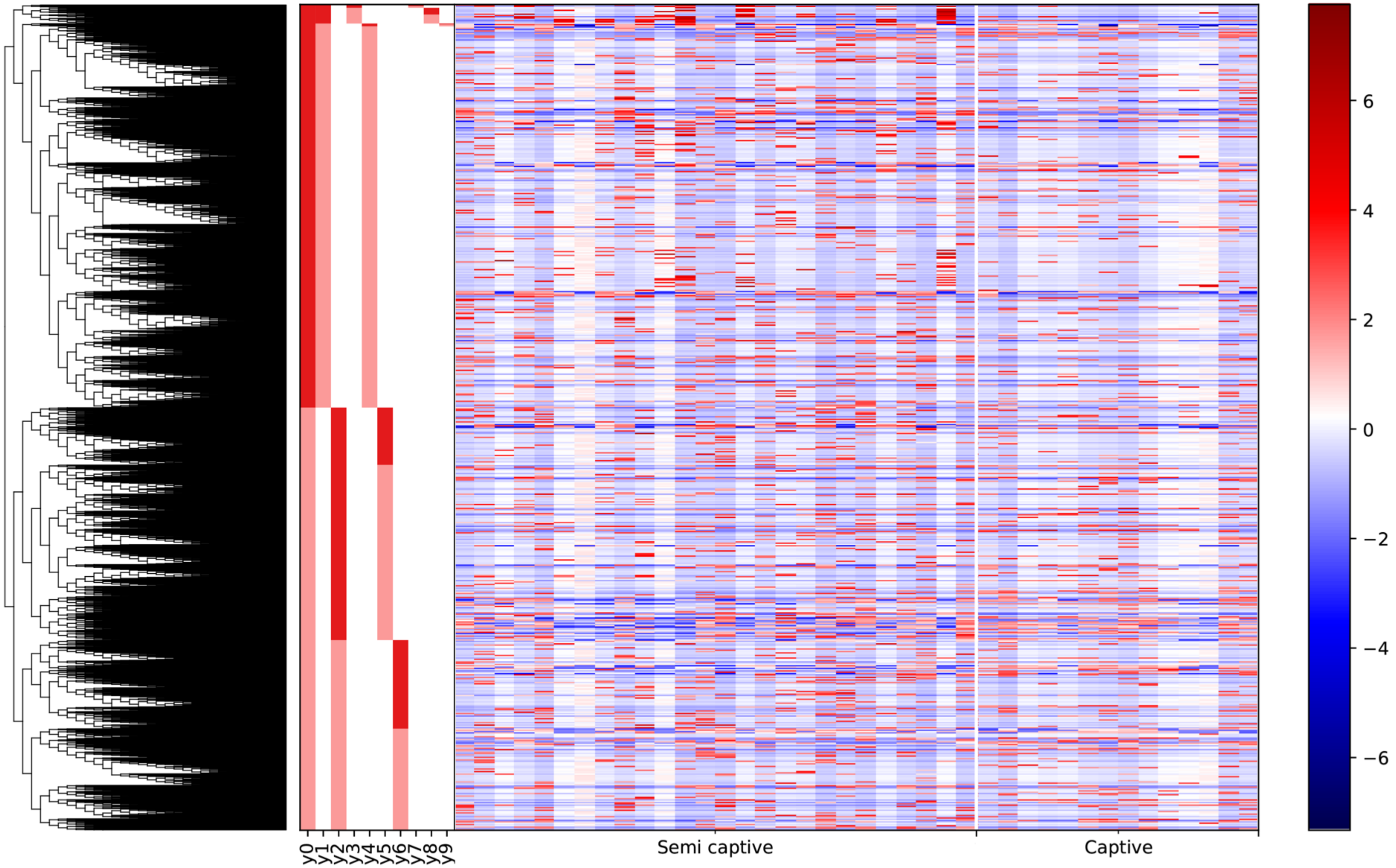

Supplementary Fig. S7 A dendrogram heatmap showing the effect of **captivity** on log abundance of ASVs in the gut microbiota of Asian elephants. Changes in the relative abundances in semi-captive and captive animals can be seen in the balance y0, y2 and y6 (gneiss analysis).

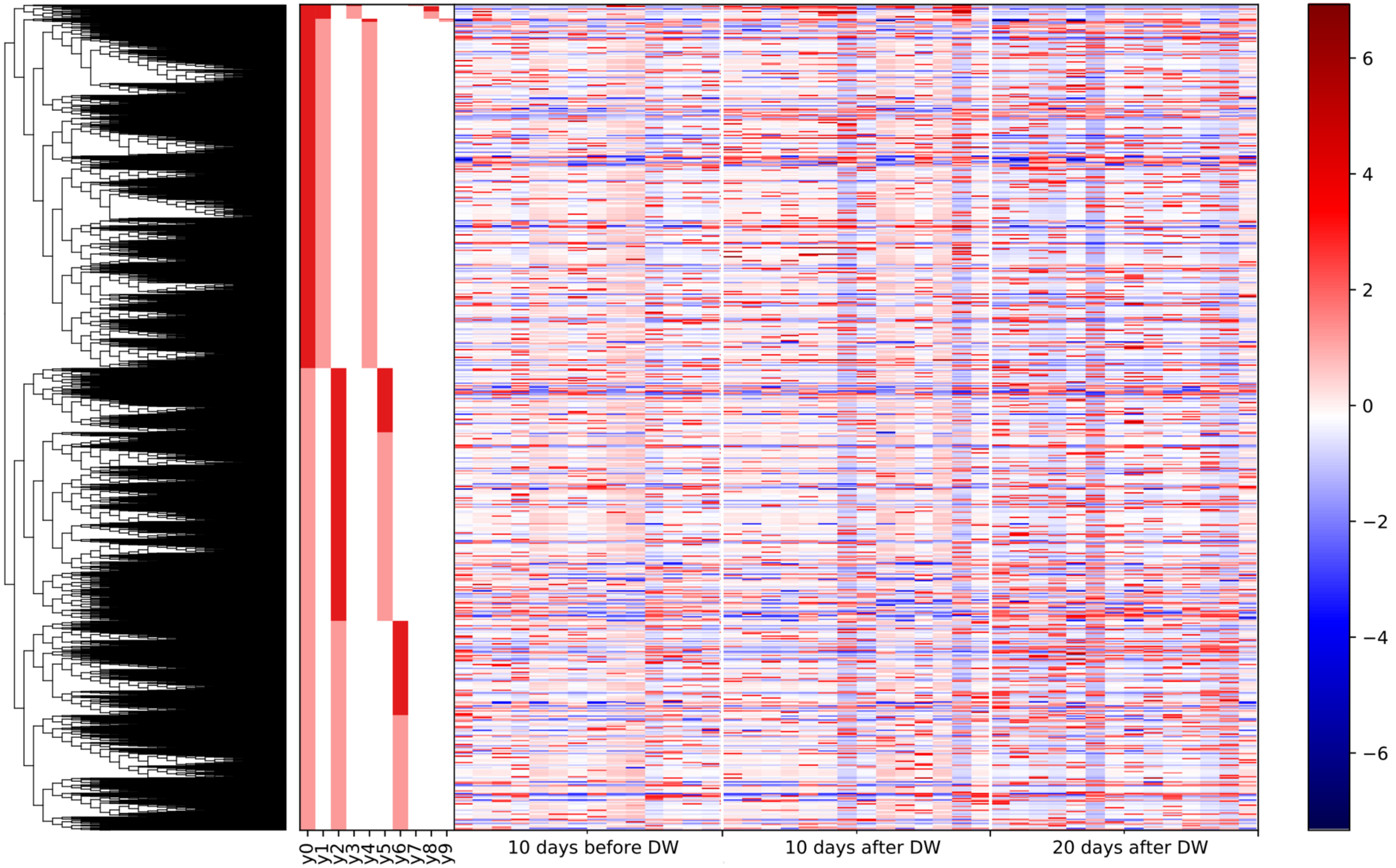

Supplementary Fig. S8 A dendrogram heatmap showing the effect of **deworming** on log abundance of ASVs in the gut microbiota of Asian elephants. Changes in the relative abundances 10 days before, 10 days after and 20 days after deworming can be seen in the balance y0, y2 and y6 (gneiss analysis).

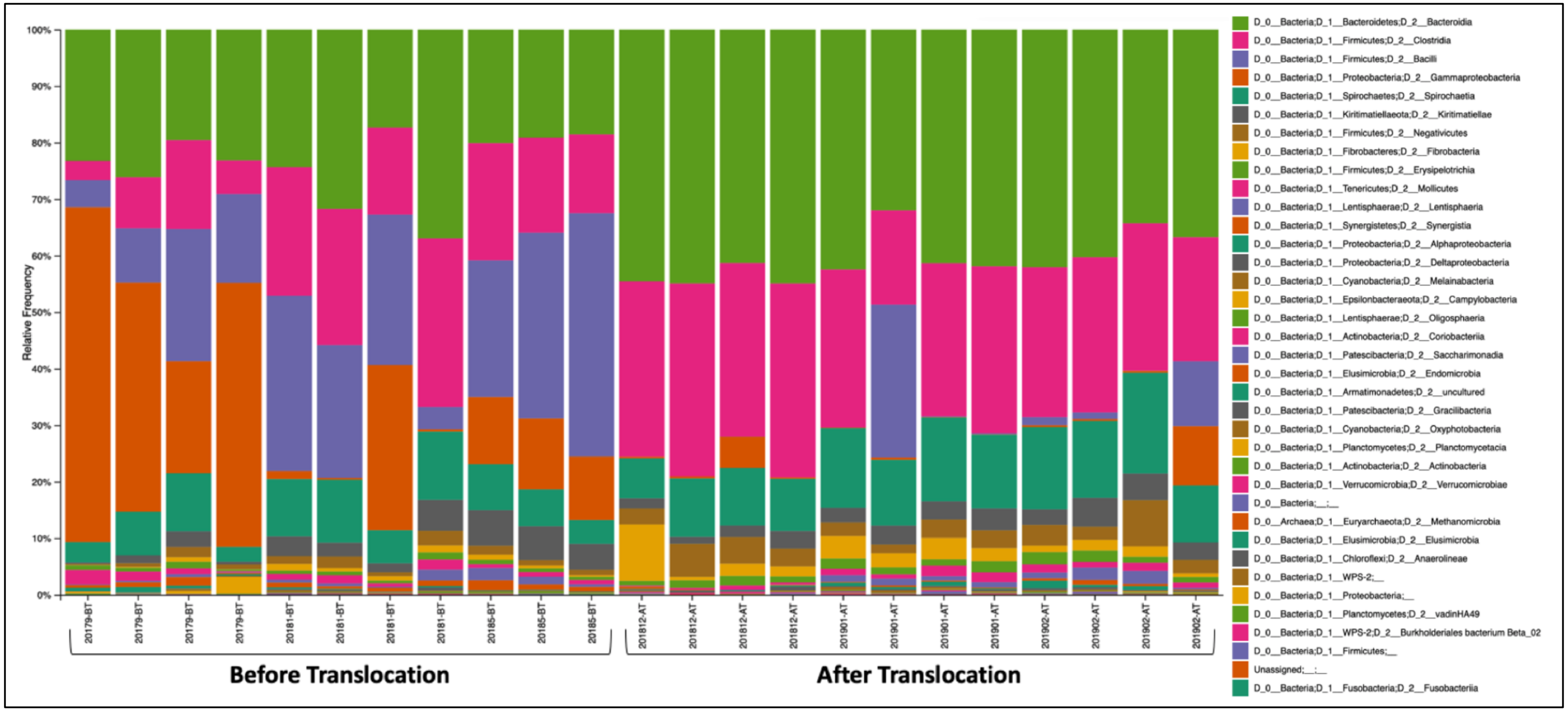

Supplementary Fig. S9 Relative abundance of taxonomic groups associated with Asian elephants in relation to Translocation. Collection dates are represented for each sample. The microbes were collapsed to the class level.

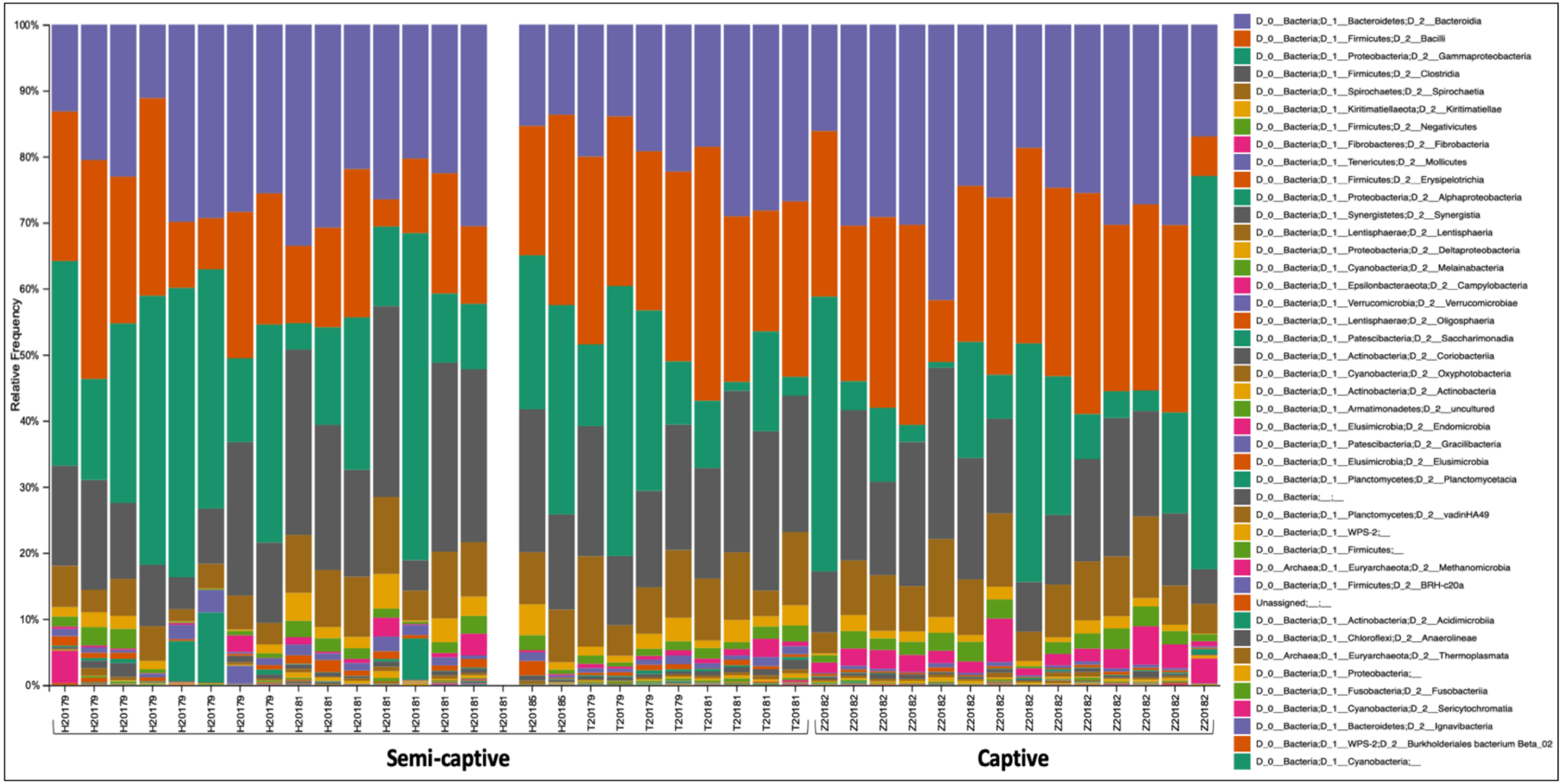

Supplementary Fig. S10 Relative abundance of taxonomic groups associated with Asian elephants in relation to captivity. Collection dates are represented for each sample. The microbes were collapsed to the class level. Sample IDs are identified by letters that describe the origin of each elephant where **(H)** represents DAA group from Hmaw Yaw Gyi camp, **(T)** represents DAA group from Taung Kya camp and **(Z)** represents elephants from Nay Pyi Taw Zoo in Myanmar.

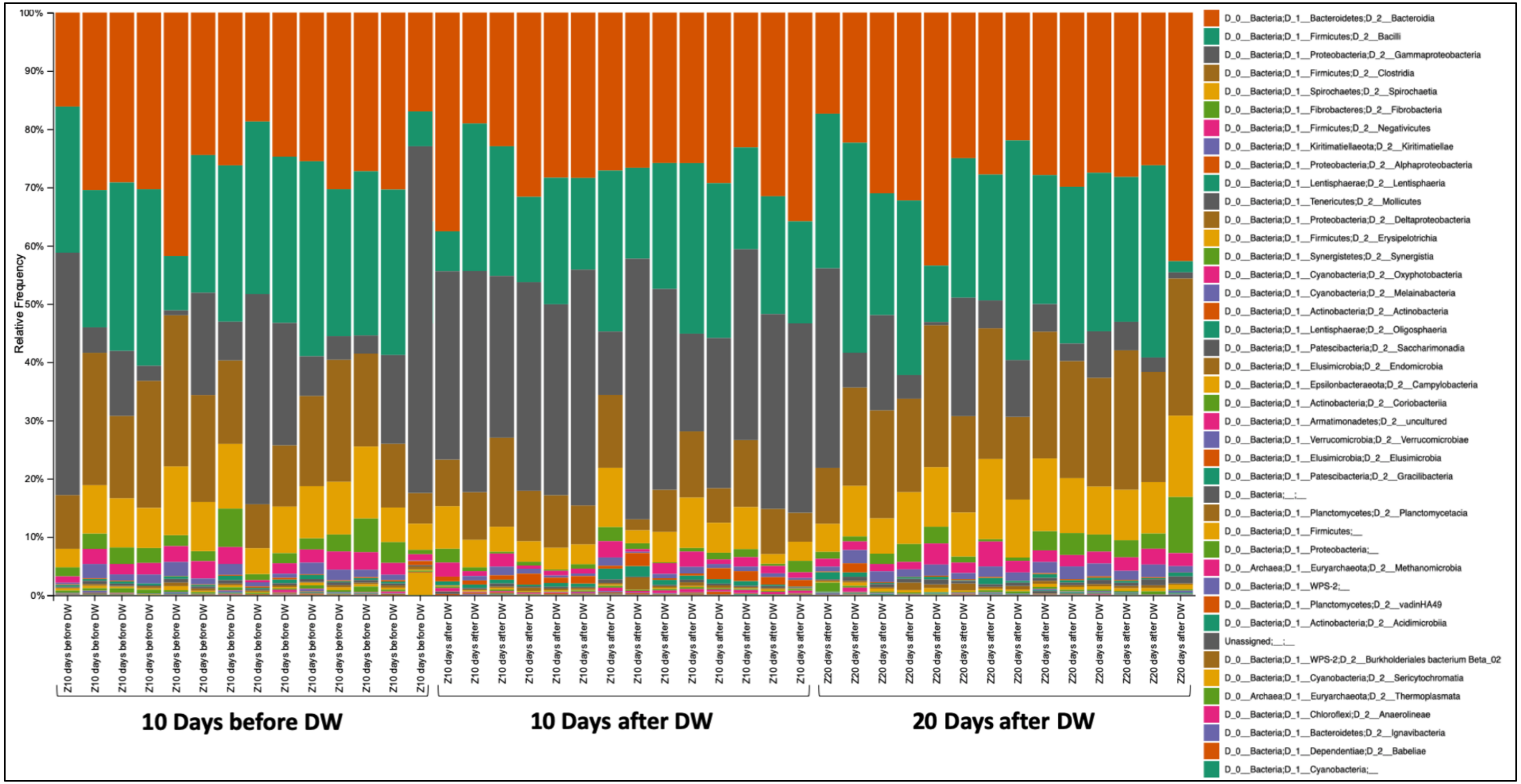

Supplementary Fig. S11 Relative abundance of taxonomic groups associated with Asian elephants in relation to deworming. The microbes were collapsed to the class level.

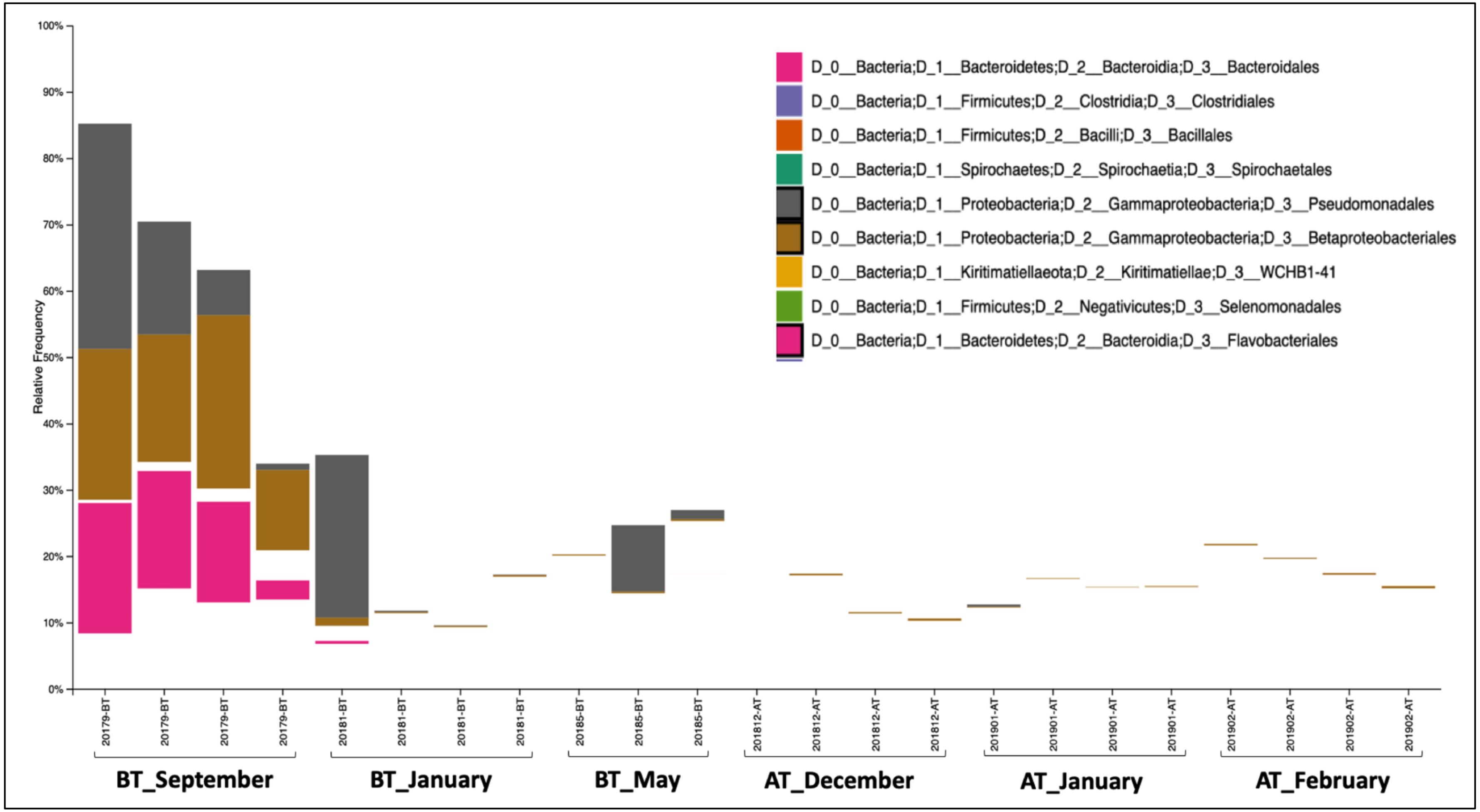

Supplementary Fig. S12 Relative abundance of Pseudomondales, Betaproteobacteriales and Flavobacteriales in the gut microbiome of Asian elephants in relation to translocation. The microbes were collapsed to the order level and showed significantly higher abundance for these three orders in September 2017.
